# Hepatitis C virus has a genetically determined lymphotropism through co-receptor B7.2

**DOI:** 10.1038/ncomms13882

**Published:** 2017-01-09

**Authors:** Chia-Lin Chen, Jeffrey Y. Huang, Chun-Hsiang Wang, Stanley M Tahara, Lin Zhou, Yasuteru Kondo, Joel Schechter, Lishan Su, Michael M C. Lai, Takaji Wakita, François-Loïc Cosset, Jae U Jung, Keigo Machida

**Affiliations:** 1 Department of Molecular Microbiology and Immunology, Keck School of Medicine, University of Southern California, 2011 Zonal Avenue, Los Angeles, California 90033, USA; 2 Department of Cell and Neurobiology, Keck School of Medicine, University of Southern California, 2011 Zonal Avenue, Los Angeles, California 90033, USA; 3 Department of Microbiology and Immunology, Lineberger Comprehensive Cancer Center, University of North Carolina at Chapel Hill, Chapel Hill, North Carolina 27599-7290, USA; 4 Institute of Molecular Biology, Academia Sinica, Taipei 115, Taiwan; 5 Department of Virology II, National Institute of Infectious Diseases, Tokyo 162-8640, Japan; 6 International Center for Infectiology Research, Team EVIR, Inserm, U1111, Université Claude Bernard Lyon 1, CNRS, UMR5308, Ecole Normale Supérieure de Lyon, Univ Lyon, F-69007 Lyon, France

## Abstract

B-cell infection by hepatitis C virus (HCV) has been a controversial topic. To examine whether HCV has a genetically determined lymphotropism through a co-receptor specific for the infection by lymphotropic HCV, we established an infectious clone and chimeric virus of hepatotropic and lymphotropic HCV strains derived from an HCV-positive B-cell lymphoma. The viral envelope and 5′-UTR sequences of the lymphotropic HCV strain were responsible for the lymphotropism. Silencing of the virus sensor, RIGI, or overexpression of microRNA-122 promoted persistent viral replication in B cells. By cDNA library screening, we identified an immune cell-specific, co-stimulatory receptor B7.2 (CD86) as a co-receptor of lymphotropic HCV. Infection of B cells by HCV inhibited the recall reaction to antigen stimulation. Together, a co-receptor B7.2 enabled lymphotropic HCV to infect memory B cells, leading to inhibition of memory B-cell function and persistent HCV infection in HCV-infected hosts.

## Abstract (web summary)

Infection of B cells by hepatitis C virus (HCV) is poorly understood, but is thought to result in lymphoproliferative disorders. Here, Chen *et al*. identify CD86 as co-receptor for lymphotropic HCV and show that HCV infection inhibits memory B-cell function.

Hepatitis C virus (HCV) infection often persists despite robust host immune responses, consequently leading to chronic hepatitis, liver cirrhosis and hepatocellular carcinoma. However, HCV infection and replication in immune cells remains controversial and is not universally accepted. Even though experimental and clinical evidence accumulated during the last two decades are compelling, the issue remains controversial mainly due to insufficient information and deeply fragmented knowledge. Another potentially serious complication of HCV infection is the possible infection of peripheral blood mononuclear cells (PBMC) by HCV leading to B-lymphocyte proliferative disorders, including mixed cryoglobulinemia, oligoclonal proliferation of B cells^1^,^2^, and B-cell non-Hodgkin's lymphoma^2^,^3^,^4^,^5^. Still, HCV infection of B cells and its possible association with B-cell disorders remains a controversial subject^6^,^7^.

It was reported from McKeating's group that HCV replication in lymphocytes is relatively rare and attachment of HCV particles to B lymphocytes did not lead to productive HCV replication^7^. HCV promoted the adhesion of primary B cells to Huh-7 cells for retention of B cells on infected hepatocytes, thus implying that B cells may provide a vehicle for HCV persistence by transmission to the liver. Additionally, lymphotropism of HCV (SB strain: patient splenoma B-cell-derived isolated by our group) is not limited to B cells since we have identified HCV infection (SB strain) of T cells and subsequent alterations in their functions^8^,^9^. These studies, however, did not provide conclusive evidence that other molecules on other immune cell types serve as HCV co-receptors.

Cellular surface receptors have been identified as factors promoting viral tropism. HCV uses cell surface factors (LDL-R and HSPG) (ref. 10) for attachment and additional entry factors for infection of hepatocytes. The entry factors include the Scavenger Receptor class B type I (SRB1 or SR-BI) (ref. 11), the tetraspanin CD81 (ref. 12), the tight junction proteins CLDN1 (ref. 13) and the receptor tyrosine kinases EGFR and EphA2 (ref. 14). More recently, the Niemann-Pick C1-like 1 (NPC1L1) cholesterol absorption receptor and the iron uptake receptor transferrin receptor 1 (TfR1) have also been shown to play a role in HCV entry^15^. Among these, four co-receptors (Claudin-1, Occludin, CD81 and SR-BI) are potentially involved in HCV entry^12^,^16^,^17^,^18^, while sulfated homologues of heparin inhibit HCV entry into mammalian cells^19^. These co-receptors are associated with the viral envelope glycoprotein of HCV. The viral envelope proteins include E1 and E2, which assemble as heterodimers in the prebudding virion form^20^.

Mutations in the 5′-UTR (5′-untranslated region) of a hepatotropic HCV strain (H77) cultured in T lymphoid cell lines enhanced viral replication specifically in T lymphoid cells (MOLT-4) (ref. 21). The presence of different, strain-specific 5′-UTR sequences or sequence heterogeneities in the region coding for E1 or E2 can result in altered lymphotropism when compared to hepatotropic strains^22^,^23^. However, the lymphotropism of these viruses and the significance of these sequence variations were not fully established since only three nucleotide substitutions found in the 5′-UTR in hepatotropic JFH1 strain and variant H77 strain passaged in lymphocytes are unchanged. The sequence variations in the 5′-UTR region are usually associated with B and T lymphocyte replication of HCV (refs 21, 24, 25). It has been shown that the B-cell specific 5′-UTR has a lower translation difference observed between lymphotropic and hepatotropic strains^26^. (The lymphotropic strain may have a less efficient 5′-UTR for translation).

The viral envelope protein is frequently mutated in chronically infected subjects, whereas the 5′-UTR of HCV RNA in B cells is not frequently mutated^22^, suggesting that B cells suppress replication of less competent viral sequences whereas liver cells are not as stringent. In SCID mice inoculated with experimentally mutated HCV strains, the HCV mutations in the 5′-UTR (specifically the IRES) or C-E1-E2 regions have different consequences on genome replication and/or translational activity as a function of cell type^26^,^27^. Moreover, the 3′-UTR also contributes to efficient RNA replication of HCV (ref. 28). The 3′-end of the NS5B-coding region has been shown to form part of a secondary structure (kissing loop) involving the 3′-UTR (ref. 28). This fact may explain why the HCV pseudoparticle (HCVpp) bearing HCV envelope alone did not confer robust replication ability in B cells.

MicroRNA-122 (miR-122) is the most abundant miRNA species in liver and binds the 5′-UTR of HCV genomic RNA to facilitate HCV replication. Chronic liver damage results in release of miR-122 into the blood stream and exposes miR-122 to immune cells. However, the role of miR-122 for HCV replication in immune cells is not documented. MicroRNA-122 is not only expressed in the liver but also in malignant and chemotherapy-treated lymphocytes^29^,^30^, and positively regulates HCV replication in hepatocytes^31^,^32^,^33^. Circulating miR-122 can be taken up by several cell types, including lymphocytes^34^,^35^ thereby facilitating HCV replication. We hypothesized that this uptake of miR-122 facilitates HCV replication in B cells. Indeed, circulating plasma miR-122 in exosomes is elevated in early stage fibrosis induced by various aetiologies, including HCV infection, hepatocyte injury and inflammation^36^,^37^,^38^,^39^.

In this study, we hypothesized that the ability of certain HCV strains to infect B cells was determined by the sequence variation of the viral genome. We used genetic approaches to show that different HCV strains vary in their relative lymphotropism or hepatotropism. The B-cell infection by lymphotropic HCV may play a role in HCV pathogenesis. These results are crucial for studying the natural history of HCV infections in order to gauge the full scope of its subsequent pathological consequences resulting from HCV infections. We investigated the roles of plasma miR-122 in HCV pathogenesis, especially in lymphocytes^31^ and the role of exosome-mediated delivery of tropic factors in creating a permissive environment for viral infection and replication.

## Results

### Lymphotropic SB-HCV strain encodes a unique E1-E2

We have previously isolated an HCV clone (SB-HCV) from an HCV-infected (+) B-cell lymphoma line^40^. This virus variant can infect B cells *in vitro*^40^; however, virus production was low for reasons unclear. To answer this question we used a genetic approach and assembled the full-length SB viral genomic RNA (genotype 2b) cloned from HCV infected SB cells^40^. Viral RNA was cloned in 14 overlapping cDNA fragments using primer sets based on the consensus sequences (Supplementary Fig. 1). Sequence determination of the resulting full-length, HCV cDNA showed that the SB-HCV strain represented a unique HCV isolate.

**Figure 1:**
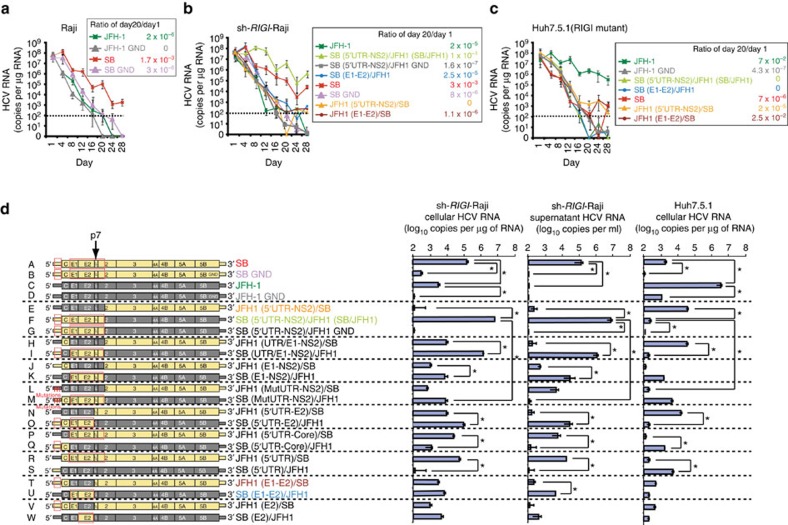
Genetic determinants of tissue tropism of HCV SB and JFH1 viruses. (**a**–**c**) HCV virus RNA in the parental Raji cells (**a**), RIG-I knockdown Raji (sh-*RIGI*-Raji) cells (**b**) or Huh7.5.1 cells (**c**). Cells were harvested from day 1 until day 28 after HCV RNA transfection. The HCV RNA titers were determined by qRT-PCR (performed in quadruplicates, *n*=3). Error bars represent s.d. (**d**) Genetic structure of virus chimera assembled from the SB and JFH-1 strains. The chimeric viruses were transfected into Raji (sh-*RIGI*-Raji) or Huh7.5.1 cells as indicated; cellular HCV RNA was quantitated by qRT-PCR at 24 days after transfection (Left and Right). The HCV RNA titers in the supernatant are shown in (**d**, middle). HCV SB, but not commonly studied JFH-1 strain, preferentially infects, replicates and produces virion particles in sh-*RIGI*-Raji lymphoma B cells, but not in Huh7.5 hepatocyte cells (*n*=3). Error bars represent s.d.

To determine whether unrelated HCV strains replicate to differing extents in B cells, we compared the full-length RNAs from the SB and JFH-1 strains, the latter of which is a well-established hepatotropic strain^41^, by *in vitro* transfection of either Huh7.5.1 cells (a hepatocellular carcinoma cell line with inactivating mutations in RIG-I) or Raji cells (a human B-lymphoid cell line) (Fig. 1). In Raji cells, the SB viral RNA titer remained at 10^4^-10^5^ copies μg^−1^ of RNA at day 20 post-transfection. For the corresponding RNA polymerase active site (GND) mutant RNA, which contains an inactivating mutation of NS5B (replication incompetent virus) (Fig. 1a), viral RNA decreased over three weeks of incubation. By comparison, the RNA titer of the JFH-1 strain quickly declined in Raji cells. These results indicated that the SB-HCV strain, but not the JFH-1 strain, was able to replicate or was unstable RNA in Raji cells. Conversely, in Huh7.5.1 cells (Fig. 1c), the SB-HCV strain was less efficiently replicated by day 28 post-transfection when compared to Raji cells (Fig. 1a); this indicated that Raji cells are more permissive for SB strain replication than Huh7.5.1. cells.

During the chronic HCV infection stage, the RIGI RNA sensor pathway is impaired because HCV protease NS3/4A cleaves the RIGI adaptor protein MAVS (IPS-1) (refs 42, 43). Furthermore, chronic inflammatory cytokines induce negative feedback pathways through induction of NLRX1 (refs 44, 45) to moderate the excessive activation of RIGI pathways. To study how HCV interacts with B cells, Raji cells were infected with a lentiviral vector expressing shRNA targeting RIGI to reduce the antiviral defences (Fig. 1b). Indeed, silencing RIGI in Raji cells significantly increased HCV RNA levels and reduced interferon (IFN)-β induction probably due to defective viral RNA recognition and induction of RIGI downstream genes (that is, IFN-β). In contrast, silencing of MDA5, another innate immune receptor, did not have any further effect (Supplementary Fig. 2), which was consistent with the finding that HCV RNA binds to RIGI, but not to MDA5 (ref. 46). The highly permissive hepatotropic liver cell line (Huh7.5.1), which is highly permissive for hepatotropic HCV, has an amino acid replacement (T55I) in the first CARD-homology domain^47^ of RIGI, resulting in inactivation of RIGI function. Therefore, the tropism of HCV-SB and hepatotropic strains was compared under identical RIGI-deficient conditions (Fig. 1b,c).

To identify the viral gene or genome region(s) responsible for the differential cellular tropism of these viral strains, we constructed chimeric virus genomes from the lymphotropic SB-HCV and hepatotropic JFH-1 HCV strains (Fig. 1d). These constructs were then converted into full-length infectious RNA for transfection into recipient cells (Fig. 1d). From the results of the various viral genome chimeras, it was apparent that all chimeric genomes containing the SB 5′-UTR and homologous structural proteins replicated well in sh-*RIGI*-Raji cells and released viral RNA (some of them are associated with virion) into the cell culture supernatant. In particular, a chimera containing the JFH1 backbone and the entire SB 5′-UTR-Core-E1-E2-p7-NS2 (construct F: at the second transmembrane domain of NS2) replicated 50-fold more robustly than the parental SB virus in sh-*RIGI*-Raji cells, producing nearly 10^7^ HCV RNA copies ml^−1^. By contrast replication-incompetent SB/JFH1 GND mutant virus RNA levels were significantly lower (construct G). The corresponding reciprocal genome constructs containing E1-E2 and the 5′-UTR from JFH1 (construct E) replicated poorly in sh-*RIGI*-Raji cells. In Huh7.5.1 cells, this result was reversed: JFH-1 RNA replicated to a level of 10^7^ RNA copies ml^−1^, whereas SB RNA replication was decreased to a significantly lower level (*P*<0.05, *t*-test). Furthermore, mutations in the 5′-UTR regions of SB and JFH1 strains (constructs L and M) resulted in significantly reduced HCV RNA levels in lymphocytes. These results suggested that there are distinct hepatotropic and lymphotropic HCV strains, whose tropism is largely determined by the envelope proteins (E1-E2) and their respective 5′-UTRs. This dramatic increase was probably due to the sequence changes that occurred at a crucial junction between the second transmembrane segments of NS2 as previously suggested^48^.

**Figure 2:**
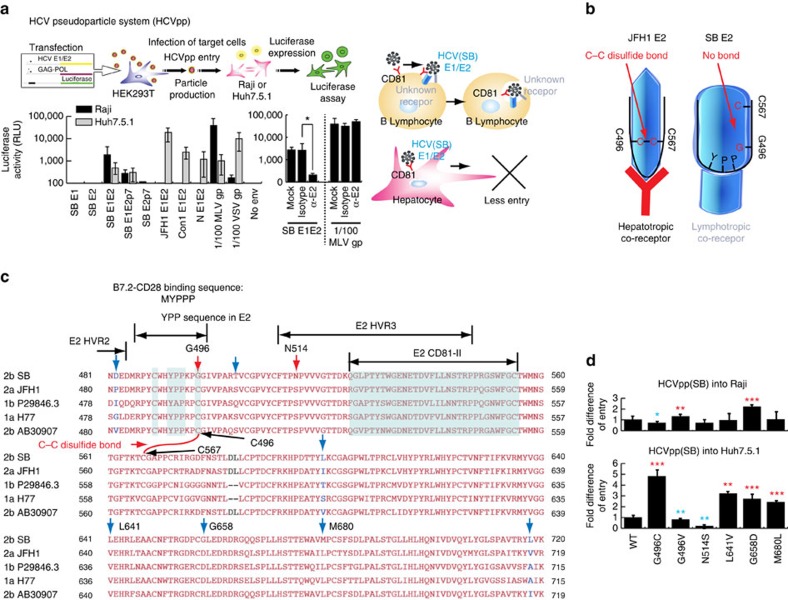
Lymphotropic pseudoparticles (HCVpp) can infect B lymphocytes more efficiently than hepatic cells. (**a**) HIV-luc vector was pseudotyped with envelope proteins of HCV SB strain. Pseudoparticles (HCVpp) efficiently infected B lymphocytes, but not hepatic cells. Infectivity of HCV pseudo-particles was measured by luciferase assay. HIV vector containing a luciferase reporter pseudotyped with E1 and/or E2 of SB strain, Con1, or HCV-N strain and MLV glycoprotein (MLV gp), or no envelope (no env) was used to infect Raji (black bars) or Huh7.5.1 (grey bars). Luciferase activity was assayed at 4 days after infection. Addition of neutralizing anti-E2 antibody blocked HCVpp entry (**a**, right). Experiments were repeated at least three times (*P<0.05, *T*-test, *n*=3). Error bars represent s.d. (**b**) Hypothetical model of E2 sequence mutations on HCV B cell tropism. (**c**) The HCV E2 amino acids sequences are variable. Red arrows indicate the different amino acid positions between SB and other HCV strains. 1b strain is from sequence accession number P29846.3. (**d**) Variations of E2 sequences change HCV tropism (*P<0.05; **P<0.01; ***P<0.001, ANOVA, *n*=3). Error bars represent s.d.

To further ascertain the importance of E1-E2 proteins in the lymphotropism of SB virus, we produced HCVpp using an envelope-defective HIV-1 proviral vector^49^,^50^,^51^. This HCVpp bears the E1 and E2 envelope proteins of HCV and contains a luciferase reporter gene in the lentivector. As shown in Fig. 2a, HCVpp bearing the SB E1-E2 envelope proteins could efficiently infect Raji cells, whereas its infectivity of Huh7.5.1 cells was significantly lower. As a control, HCVpp assembled with either E1 or E2 proteins alone were not infectious. The infectivity of HCVpp with SB envelope protein in Raji cells was substantially lower (more than 3-logs lower) than that of HIV vector pseudotyped with MLV Env. Addition of neutralizing anti-E2 antibody (CBH5 clone) specifically antagonized HCVpp entry into Raji cells (Fig. 2a, right). The ability of HCVpp-SB to infect Raji cells gives further support that the SB strain is lymphotropic while HCVpp-JFH1 do not infect Raji cells. Indeed, in the CD81-interacting domain of the E2 region^52^,^53^,^54^, the SB-HCV amino acid sequence is homologous to other HCV strains, but does have several unique residues that may be responsible for the observed lymphotropism (Fig. 2c). Another genotype 2b strain (AB 30907) has the highest homology to the SB strain among all HCV genotypes while the H77 genotype 1 strain has lesser homology with the SB strain (Fig. 2b,c). Amino acids G496 and N514 of SB HCV E2 are unique amino acids in comparison to other HCV E2 sequences, which contain S514 (ref. 55). The G496C substitution has been shown to form a disulfide bond with C567 (ref. 55). Substitution of G496 by Cys significantly reduced SB E2 binding to B cells while this substitution significantly increased virus entry into Huh7 cells (Fig. 2d). Formation of the disulfide bonding between G496C and C567 may restore or stabilize a conformation favourable of E2 for entry into hepatocyte HCV co-receptors (Fig. 2d). These results confirmed that the E1-E2 envelope proteins conferred the observed lymphotropism exhibited by the SB strain in addition to the properties of strain-specific 5′-UTRs.

### Envelope and 5′-UTR determine lymphotropism of SB virus

**Figure 3:**
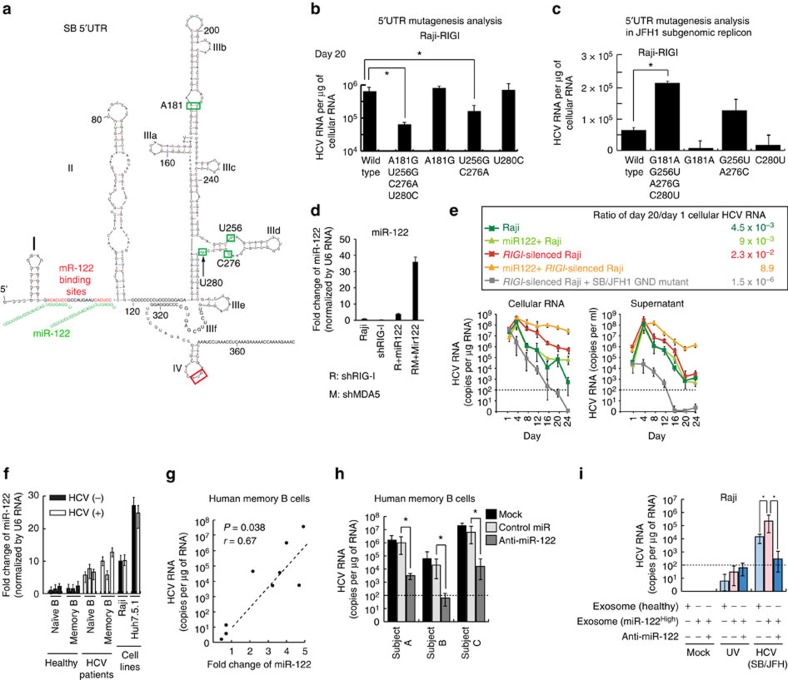
Sequence of 5′-UTR of lymphotropic HCV and cellular miR-122 levels are involved in lymphotropism. (**a**) The putative structure of the 5′-UTR of SB-HCV RNA as determined by MFOLD program (computational secondary structure prediction software). The nucleotides in green boxes are those that differ between SB and JFH1 RNA. These nucleotides are located in stem-loops (IIIa and IIId) and bulge regions, which may recruit lymphocyte-specific factors. The nucleotides in red boxes are the first AUG (start codon). miR-122 seed-match sequences (red) and predicted interactions with miR-122 (green) are shown. (**b**) The HCV RNA titers in sh-RIGI-Raji cells transfected with various 5′-UTR mutants of SB RNA (*P<0.05, ANOVA, *n*=3). Error bars represent s.d. (**c**) The HCV RNA titers in sh-RIGI-Raji cells transfected with the various 5′-UTR mutants of JFH1 subgenomic replicon RNA (*P<0.05, ANOVA, *n*=3). Error bars represent s.d. (**d**) miR-122 expression levels in Raji cells transduced with lentivirus expressing shRIGI, sh-MDA5 or mir-122 (*n*=3). Error bars represent s.d. (**e**) Transduction of microRNA miR-122 enhances and stabilizes HCV replication in B lymphocytes. Effects of RIGI and miR-122 on lymphotropic HCV SB infection were examined using specific shRNA (*n*=3). Error bars represent s.d. (**f**) Human memory B cells have higher levels of miR-122 in clinical specimens. Cellular miR-122 levels were determined by qRT-PCR in quadruplicate in PBMCs from three different patients (*n*=3). Error bars represent s.d. (**g**) Analysis of HCV RNA in B cells versus miR-122 in B cells. The miR-122 levels of B cells from HCV patients were comparable to that of Raji cells. The miR-122 abundance is correlated with higher HCV RNA levels in memory B cells. Cellular HCV RNA levels were determined by qRT-PCR in nine different patient PBMCs. (**h**) Memory B cells from three patients were infected with HCV and cellular HCV RNA levels were determined by qRT-PCR in quadruplicate. (*P<0.05, Student *t*-test, *n*=3). Error bars represent s.d. (**i**) To determine if miR-122 sequestration reduced HCV replication in B cells, antagomir of miR-122 was transfected into human memory B cells isolated from HCV patients and examined for cellular levels of HCV RNA in B cells (*P<0.05, *t*-test, *n*=3). Error bars represent s.d.

The predicted secondary structure of the SB 5′ UTR includes several possible RNA stem-loop structures (Fig. 3a). Five nucleotide differences between the SB and JFH1 strains are found within 5′-UTR and their positions are indicated (Fig. 3a). To address the significance of these genetic differences, and potential effects on RNA secondary structure, four out of five nucleotides were changed from the SB strain to those found in the JFH1 strain. Interestingly, the four-nucleotide substitutions significantly reduced HCV RNA levels in sh-*RIGI*-Raji cells at 20 days post transfection of HCV RNA (Fig. 3b). The A181G and U280C substitutions did not affect HCV RNA levels, but combined substitutions (A181G, U256G, C276A and U280C), and to a lesser extent, U256G and C276A substitutions reduced HCV RNA levels. However, it cannot be excluded that these differences may be due to changes in RNA stability. Therefore, we constructed a mutant subgenomic JFH1 replicon to see if substitutions of the 5′-UTR sequence promoted replication in B cells. The 5′-UTR sequences were replaced with SB 5′-UTR sequences of the JFH1 subgenomic HCV replicon. We observed that combined substitutions in G181A, G256U, A276C and C280U increased HCV RNA levels of the JFH1 subgenomic replicon (Fig. 3c) in sh-*RIGI*-Raji cells. These results indicated that nucleotides A181, U256, C276 and U280 of the 5′-UTR have key roles in regulating HCV levels in sh-*RIGI*-Raji cells.

To prove that SB-HCV recombinants produced infectious virus particles, the culture supernatant from the transfected cells was used to infect naïve sh-*RIGI-*Raji or Huh7.5.1 cells by using a batch system to ensure a cell-free transfer (Supplementary Fig. 3). SB RNA-transfected Raji cells produced infectious virus that infected naïve sh-*RIGI*-Raji cells (Supplementary Fig. 3A and B), but was less efficient at replication in Huh7.5.1 cells (Supplementary Fig. 3C). The converse was true for the JFH strain in Huh7.5.1 cells (Supplementary Fig. 3C). Infectivity of SB/JFH1 virus was directly quantified by limiting dilution assay, demonstrating that HCV-infected cells have infectivity of 40% (6/15 clones) at day 13 and 38% (6/16 clones) at day 20 (Supplementary Fig. 3D). Infection of HCV did not significantly affect cell proliferation (Supplementary Fig. 3E).

The role of miR-122 in HCV lymphocyte infection was examined by transduction of miR-122 into B lymphocytes. A miR-122 expressing lentivirus was transduced into sh-*RIGI*-Raji cells and cells were challenged with HCV (Fig. 3d). The results showed that miR-122 transduction significantly enhanced HCV replication, producing virus titers of 2 × 10^6^ copies ml^−1^ and 10^8^ copies μg^−1^ of cellular RNA (387-fold increase: *P*<0.001, *t*-test) after 3 weeks post-transduction (Fig. 3e). Cellular miR-122 levels were significantly reduced 4 days post-transduction while levels of miR-122 significantly increased in the supernatant (Supplementary Fig. 4). Indeed, HCV RNA levels in culture supernatant significantly increased by day 7 and further increased at day 14 post-transfection (*P*<0.05, *t*-test, Supplementary Fig. 4, right), indicating that miR-122 may be secreted from HCV-infected B cells. Furthermore, miR-122 levels were measured in immune cells isolated from HCV patients. Consistent with *in vitro* results, circulating miR-122 levels were significantly increased in HCV-infected patients (*P*<0.05, *t*-test, Fig. 3f). The increased expression of miR-122 in B cells was confirmed in memory B cells from HCV patients (*n*=9). The higher miR-122 levels in memory B cells showed a positive correlation with higher cellular HCV RNA levels (*r*=0.67, *P*<0.05, *t*-test, Fig. 3g).

In order to determine if reduction of miR-122 levels had a negative effect on HCV replication in B cells, an antagomir of miR-122 was transfected into human memory B cells isolated from HCV patients and examined for effect on cellular levels of HCV RNA in B cells. These primary cells were incubated *ex vivo* to allow HCV replication. Interestingly, transfection of miR-122 antagomir into patients' memory B cells significantly lowered HCV replication (*P*<0.05, *t*-test; Fig. 3h).

Transfer of miR-122 via exosomes was tested *in vitro* for effect on HCV replication. Exosomes derived from HCV patients or healthy individuals were added to the supernatant of HCV-infected Raji cells and examined for effect on HCV replication. Addition of exosomes containing high miR-122 levels from HCV patients indeed enhanced HCV replication (14-fold increase of HCV RNA) in Raji cells (Fig. 3i). These results indicated that miR-122 promotes HCV replication in B lymphocytes.

### CLDN1/OCLN can rescue hepatotropic HCV infection

**Figure 4:**
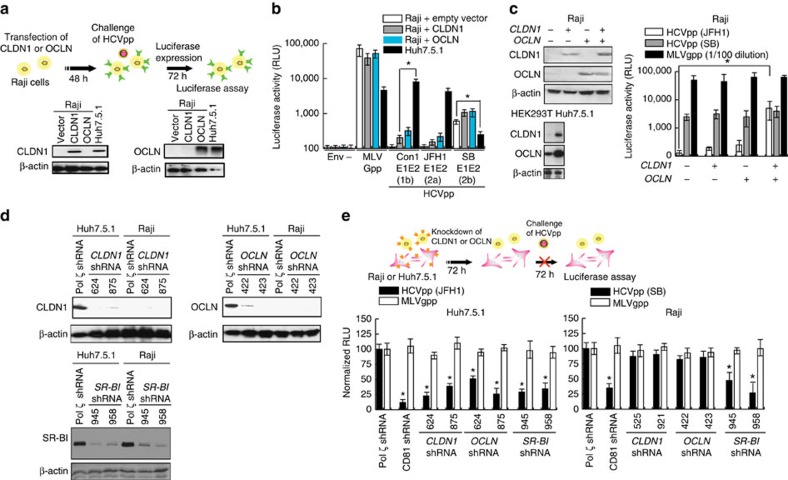
HCV pseudo-particles (HCVpp) with envelope proteins of SB strain infected B lymphocytes, but not hepatic cells. (**a**) Claudin1 (CLDN1) or Occuludin (OCLN) expression confers weak susceptibility of Raji cells to JFH1 infection (Upper panel). Immunoblot analysis demonstrated that Claudin-1 and Occludin proteins were expressed by transfection of plasmids. (**b**) The respective expression vectors of CLDN1 and OCLN were transfected into both Raji and Huh7.5.1 cells. Luciferase-encoding pseudo-particles bearing the indicated envelope proteins were used to infect recipient Raji cells transfected with control vector (white), CLDN1-expressing vector (grey), or OCLN-expressing vector, or control Huh7.5.1 cells (black). For HCVpp, the respective E1E2 genes are indicated in parentheses. Luciferase assay was performed at 3 days after transduction (*P<0.05, *t*-test). Values are normalized to the RLU background measured in mock-infected cells (mean of *n*=3; error bars, s.d.). RLU: relative luminescence units. (**c**) The respective expression vectors of CLDN1 and OCLN were transfected and their protein expression levels were confirmed in both Raji and Huh7.5.1 cells. (*Left panel*) Expression of both CLDN1 and OCLN (tight junction molecules) promoted HCVpp(SB) entry (right panel) (*P<0.05, *t*-test, *n*=3). Error bars represent s.d. (**d**) Cells were transfected to express shRNA against CLDN1 or OCLN and examined for silencing effects by immunoblots. shRNAs against DNA polymerase *ζ* (an irrelevant shRNA for HCV entry) were used as the negative and positive controls, respectively. Two different shRNA clones targeting either CLDN1 or OCLN were used. (**e**) Silencing of tight junction molecules CLDN1 and OCLN did not inhibit HCVpp(SB) entry in lymphocytes while expression of CLDN1 and OCLN is required for the susceptibility to HCVpp(JFH1) in hepatocytes. Cells were challenged with HCVpp (black bars: pseudotyped with SB or JFH1 E1E2) or MLV-Gpp (white bars) encoding a luciferase reporter (*P<0.05, *t*-test, *n*=3). Error bars represent s.d.

We next studied the cellular basis for the lymphotropism of the SB virus by focusing on the role of CLDN1 and OCLN, which have been shown to be the co-receptors for HCV infection of Huh7.5.1 cells^13^,^16^. CLDN1 and OCLN are normally expressed at very low or undetectable levels in Raji cells (Fig. 4a) (ref. 56). To test whether overexpression of either CLDN1 or OCLN could render Raji cells more susceptible to infection by hepatotropic JFH-1, we overexpressed CLDN1 or OCLN in Raji cells. As shown in Fig. 4b, overexpression of CLDN1 or OCLN in Raji cells resulted in only a marginal increase of viral entry and not to the extent observed for SB (E1-E2). By comparison, HCVpp bearing E1-E2 of Con1 or JFH-1 could not infect Raji cells. It is not clear if lower infectivity was due to inability to adsorb and enter cells or due to lower efficiency of viral gene expression and replication. To test if HCV cell entry required both OCLN and CLDN1 co-receptors, both OCLN and CLDN1 were simultaneously transfected into Raji cells prior to challenge with HCVpp-JFH1. The extent of successful HCV intracellular entry was assessed by luciferase assay (Fig. 4c). As four human co-receptors (OCLN, CLDN1, CD81 and SR-BI) are critical for HCV entry into hepatocytes, the effect of OCLN and CLDN1 overexpression was examined in Raji cells, which normally lack their expression. Interestingly, Raji cells expressing both OCLN and CLDN1 were found to promote entry of hepatotropic JFH1 HCV (Fig. 4c), confirming that viral entry required co-expression of both OCLN and CLDN1. The intrinsic ability of HCVpp-SB to infect Raji cells, but not HCVpp-JFH1, in the absence of OCLN and CLDN1, to infect Raji cells gives further support to our contention that the SB strain is lymphotropic (while Huh7 cells express all four receptors CLDN1, OCLN, CD81 and SR-BI).

On the other hand, CLDN1, OCLN, SR-BI or CD81 knockdown in Huh7.5.1 cells potently inhibited the infection by HCVpp-JFH-1 (Fig. 4d,e), confirming the previous report describing essential roles of four co-receptors CLDN1, OCLN, CD81 and SR-BI for virus entry into hepatocytes^12^,^13^,^16^,^57^. In contrast, while the knockdown of CD81 or SR-BI inhibited HCVpp-SB infectivity in Raji cells, knockdown of CLDN1 or OCLN did not exhibit this inhibition. The results of Fig. 4d,e indicated that, in contrast to JFH1, SB strain entry is a CD81- and SR-BI-dependent but CLDN1- and OCLN-independent pathway for infection of B cells.

### B7.2 is a co-receptor for lymphotropic HCV of B cells

**Figure 5:**
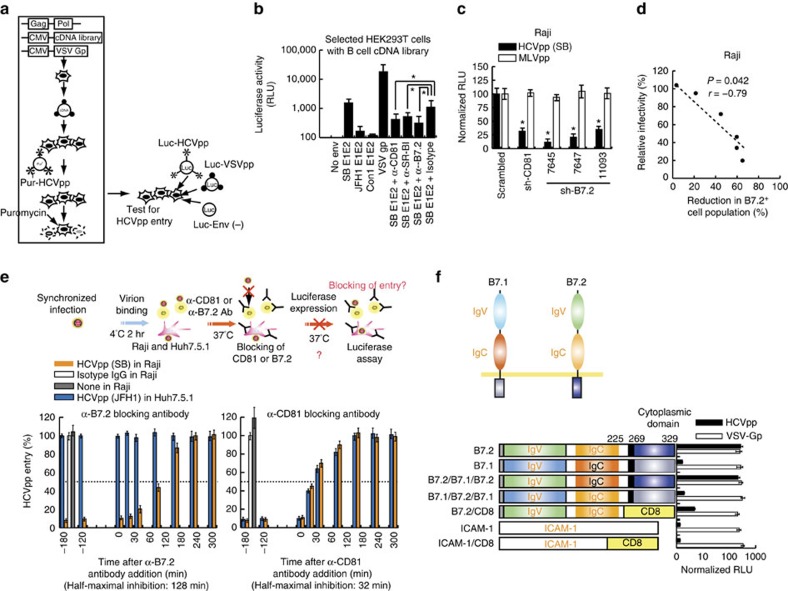
Co-stimulatory molecule B7.2/CD86 is identified as a co-receptor for lymphotropic HCV infection in B lymphocytes. (**a**) Expression cloning of lymphotropic HCV receptor. HEK293T cells were transfected with B-cell cDNA library and selected with functional screening using HCVpp(SB) as the ligand and puromycin or other selection antibiotic. To recover B-cell cDNAs in selected (surviving) colonies, DNA was isolated from these selected HEK293T cells and amplified by PCR with specific primers to identify the inserted cDNA library sequence. (**b**) The blocking effects of anti-B7.2 and anti-CD81 on HCVpp(SB) infection (*P<0.05, *t*-test, *n*=3). Error bars represent s.d. (**c**) B7.2 silencing inhibited HCVpp-SB entry into Raji cells. The HCVpp entry experiment was performed in triplicate (*n*=3). Error bars represent s.d. (**d**) Correlation plot between B7.2 levels and relative infectivity. (**e**) Time course of the blocking effects of anti-B7.2 and anti-CD81 on HCVpp(SB) infection. Synchronized infections were performed on Raji or Huh7.5.1 cells. Values (percent entry) are relative to the signal seen when the antibody was added at 4 h post temperature shift. Cellular HCV RNA levels were detected in Raji cell cultures treated with isotype-matched or anti-B7.2 or anti-CD81 antibody. Fits of *t*=0 and later data points represent a one-phase exponential association and sigmoidal dose-response (variable slope) for anti-B7.2 (50 μg ml^−1^) or anti-CD81 (50 μg ml^−1^) antibody (mean of *n*=3; error bars, s.d.). (**f**) Both B7.2 IgV and cytoplasmic regions are required for efficient HCV entry. The Raji cells (B7.2^low^) transduced with lentivirus expressing sh-B7.2 were electroporated with GFP-B7.2 (B7.2), GFP-B7.2/CD8 (B7.2/CD8), GFP-ICAM-1 (ICAM-1) or GFP-ICAM-1/CD8 (ICAM-1/CD8) vectors. HCV entry levels were assessed for luciferase activities at 72 h after HCVpp challenge of transfected cells (*n*=3). Error bars represent s.d.

Inability of SB to infect hepatocytes indicated that some other cell surface co-receptor proteins were needed. In an effort to identify the potential novel co-receptor specific for lymphotropic HCV infection, we performed a lentivirus-based screen of a cDNA library, derived from the highly HCV-permissive B lymphoma cell lines Raji and Daudi. This screen was designed for identification of genes that rendered the non-permissive CD81^+^ SR-BI^+^ HEK293T cell line susceptible to infection by HIV-luc reporter vector pseudotyped with HCVgp (HCVpp) (Fig. 5a). The result of this functional screen identified ten co-receptor candidate genes (Supplementary Fig. 5), which were tested in shRNA-transduced Raji cells for HCVpp entry. As a result of this screen and confirmatory knockdown experiments, B7.2 (or CD86) was identified as a potential entry factor. B7.2 did not affect HEK293T susceptibility to HIV-luc particles either lacking envelope proteins (Env^−^pp) or pseudotyped with unrelated vesicular stomatitis virus G protein (VSV-Gp); these served as negative and positive infection controls, respectively. Accordingly, HCVpp-E1-E2 (SB strain) infection of Raji cells was inhibited by anti-B7.2 and anti-CD81/anti-SR-BI (known HCV co-receptors), but not by a control isotype antibody (Fig. 5b).

To further determine if B7.2 is required for HCV-SB infection of B cells, Raji cells were infected with lentiviruses expressing shRNAs targeting different regions of B7.2. The B7.2 knockdown results showed that HCVpp-SB infection was markedly reduced in B7.2-silenced cells as compared to cells treated with a scrambled shRNA (Fig. 5c). When using distinct shRNAs of variable potency against B7.2, HCVpp infectivity was inversely correlated to the degree of silencing (Fig. 5d). By comparison, B7.2 knockdown in Huh7.5.1 cells did not inhibit HCVpp (JFH1) infection (Supplementary Fig. 5A). Next, to determine if the entry step of virus infection required B7.2, synchronized infections were performed on Raji or Huh7.5.1 cells in the presence of blocking antibodies (Fig. 5e). The kinetics of inhibition by anti-B7.2 antibody was significantly slower than that for anti-CD81 antibody, with half-maximal inhibition observed at 128 min post-temperature shift while the half-maximal inhibition time of anti-CD81 was 32 min. In contrast, anti-B7.2 antibody did not inhibit HCVpp (JFH1) infection of Huh7.5.1 cells. The time course of inhibition was similar to that observed for the Claudin-1 antibody^16^. Thus, these results were consistent with the interpretation that anti-B7.2 antibody inhibited a step after virus binding.

**Figure 6:**
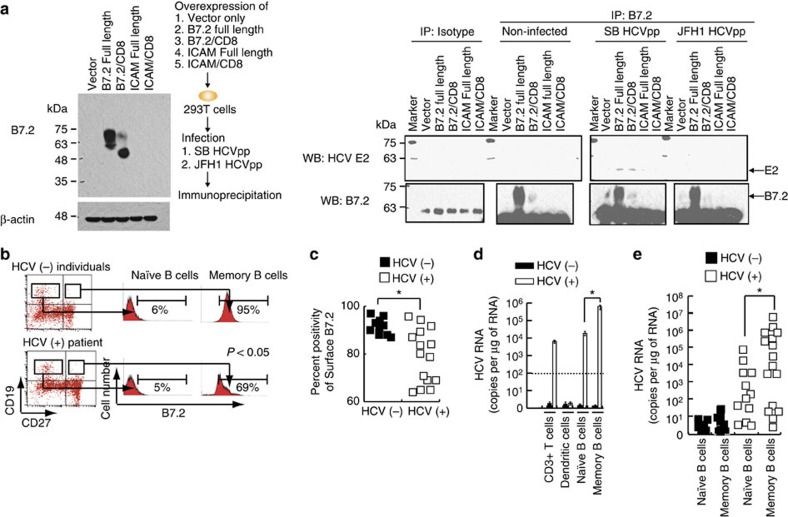
Envelope protein E2 of HCV SB strain interacts with B7.2 protein in shRIGI-Raji cells and surface B7.2 is reduced in memory B cells of HCV patients. (**a**) Envelope protein E2 of HCV SB strain interacted with B7.2 protein in shRIGI-Raji cells. Arrows indicate bands of HCV E2. (**b**) B cells from healthy individuals (upper panel) and HCV patients (lower panel) were sorted by flow cytometry into CD19+CD27- naïve B cells or CD19+CD27+ memory B cells. The former expressed a low level of B7.2, whereas the latter expressed a high level of B7.2. (**c**) Surface B7.2 expression is reduced in human memory B cells in HCV patients. Human patient B cells were sorted into two fractions, naïve B cells (CD19+/CD27−) and memory B (CD19+/CD27+), and used for RNA isolation. (**d**,**e**) Memory B cells were infected with HCV. Human memory B cells showed higher levels of HCV RNA in clinical specimens (*P<0.05, *t*-test, *n*=3). Error bars represent s.d. Analysis of HCV RNA in B cells, T cells, monocytes and dendritic cells.

We next examined which domains of B7.2 are responsible for interaction with SB envelope proteins. While exogenous B7.2 allowed HCVpp to infect B7.2-silenced Raji cells, neither B7.1 nor ICAM supported HCVpp infection (Fig. 5f). Furthermore replacement of the IgV domain of B7.2 with that from B7.1 abrogated the HCVpp infection of B7.2-silenced Raji cells (Fig. 5f). The importance of the B7.2 cytoplasmic domain was underscored since replacement of this domain with that from CD8 also resulted in the loss of HCVpp infectivity. To test if a direct interaction occurred between E2 and B7.2, we performed coimmunoprecipitation with anti-B7.2, followed by immunoblot for E2 proteins and showed an interaction between B7.2 and HCV E2 proteins (Fig. 6a and Supplementary Fig. 9). Reciprocal immunochemical analysis further confirmed E2-B7.2 interactions (Supplementary Fig. 5C and Supplementary Fig. 9). These results indicated that B7.2 is a co-receptor for HCV-SB infection of Raji cells.

We further examined whether the level of B7.2 expression in B cells of hepatitis C patients correlated with the extent of HCV infection. Naïve B cells (CD19^+^CD27^−^) were found to have low levels of B7.2 expression, while memory B cells (CD19^+^CD27^+^) showed higher expression levels (Fig. 6b), in agreement with previous reports^58^. Interestingly, memory B cells in HCV patient-derived PBMCs expressed significantly lower levels of surface B7.2 when compared to those in HCV (−) individuals (Fig. 6c). Significantly, HCV RNA levels were two logs higher in memory B cells than in naïve B cells isolated from HCV patients, which is correlated with increased protein levels of B7.2 (Fig. 6d). These findings were confirmed by examination of 18 different hepatitis C patients (Fig. 6e). Lower surface B7.2 levels were correlated with lower cellular HCV RNA levels (Supplementary Fig. 5D). Activation of B cells (CD40L+IL-4 treatment) is known to promote surface B7.2 expression in naïve B cells^59^. Interestingly, activation of B cells resulted in higher levels of cellular HCV RNA 8 days post-HCV (SB/JFH1) infection (Supplementary Fig. 5E). Anti-CD81, anti-SR-BI or anti-E2 neutralizing antibody treatment blocked HCV infection in sh-*RIGI*-Raji cells (Supplementary Fig. 5F). B7.2 expression in B and T cells and dendritic cells was confirmed by immunoblot (Supplementary Fig. 5G). These results indicated that CD81, SR-BI and E2 are involved in critical steps for lymphotropic HCV entry process into B cells. This conclusion is limited to B cells, but not in T-cell lines since SR-B1 involvement is known for HCV infection of primary T cells. Interestingly, primary T lymphocytes do not display detectable levels of SR-B1 protein, although T-cell lines do^60^.

### Characterization of HCV particles produced from SB strain

**Figure 7:**
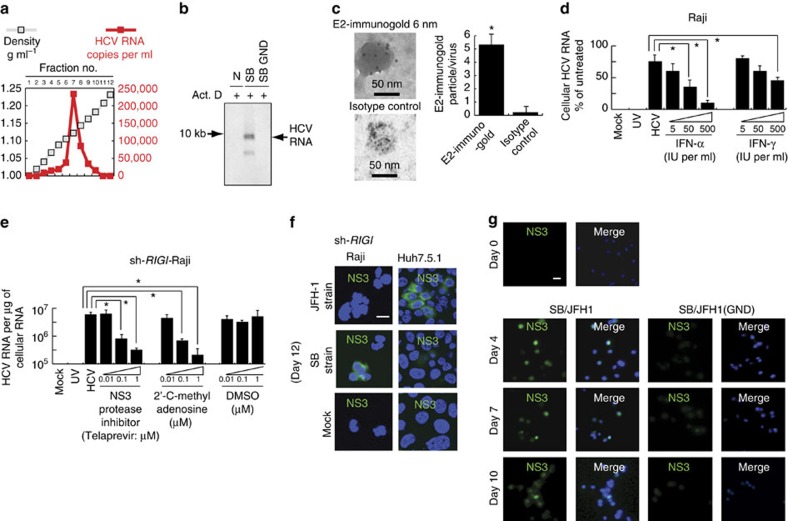
The two HCV strains SB and JFH-1 have profoundly different tissue-specificity. (**a**) Sucrose gradient sedimentation of the supernatant from the transfected cells. Concentrated culture medium collected from SB/JFH1 chimera JFH (SB5′-UTR-NS2) RNA-transfected cells was fractionated using a 20-60% sucrose density gradient. HCV RNA titer in each fraction was determined by RT-PCR. (**b**) JFH (SB5′-UTR-NS2)-transfected Raji cells were labelled with [^3^H]uridine for 16 h in the presence of actinomycin D. The supernatant RNA was concentrated and analysed by denaturing agarose gel electrophoresis. Radiolabelled RNAs were visualized with a BAS-2500 Bio-Imager (Fuji). (**c**) Electron micrograph of the SB virus concentrated by sucrose gradient fractionation. The virus was stained with anti-E2 or isotype antibody and Protein A-conjugated immunogold. Asterisk represents statistical significance (*P*<0.05, *t*-test, *n*=3). Error bars represent s.d. (**d**) Inhibition of HCV infection by IFN. Raji cells were treated with 5, 50 and 500 units ml^−1^ human IFN-α and IFN-γ (PBL Biomedical Laboratory, Piscataway, NJ, USA) for 6 h and then inoculated with SB virus in the presence of the same doses of IFN. The viral inoculum was removed 4 h later, and the cells were further cultured with IFN for 8 days. Cellular HCV RNA was isolated and analysed by real-time RT-PCR (*P<0.05, *t*-test, *n*=3). Error bars represent s.d. (**e**) SB-infected Raji cells (sh-*RIGI*-Raji) were treated with an NS3 protease inhibitor (Telaprevir) or 2′-modified nucleosides as inhibitors of HCV replication (2′-C-methy adenosine) (*P<0.05, *t*-test, *n*=3). Error bars represent s.d. (**f**) Immunofluorescence of NS3 in cells infected with SB or JFH1 RNA. Staining was done at 12 days post-infection. Green (NS3), Blue: DAPI staining. Scale bar represents 10 μm. (**g**) Time course of NS3-positive B cells after HCV infection. Scale bar represents 10 μm.

The concentrated virus particles had a density of 1.13 g ml^−1^ in sucrose (Fig. 7a), which is in agreement with previous report (1.12 g ml^−1^) of HCV virions isolated from the human T- and B-cell lines HPBMa10-2 and Daudi^25^, but slightly less than that of HCV virions isolated from hepatocytes (ca. 1.15 g ml^−1^) (refs 41, 61). To further characterize SB viral RNA, SB-transfected cells were metabolically labelled with [^3^H]uridine in the presence of actinomycin D. The viral particles produced from transfected cells were concentrated from the culture supernatant and separated by sucrose gradient sedimentation. A specific ^3^H-uridine-labelled peak, which was resistant to protease treatment, was detected in the culture supernatant of Raji cells (Fig. 7a). RNA isolated from this material yielded a major RNA species of approximately 9.7 kb and a minor RNA species. The latter could be defective interfering RNA (DI RNA), although further work will be needed to ascertain this (Fig. 7b).

Electron microscopy demonstrated that the particles were approximately 50-60 nm in diameter and possessed an envelope that was immunoreactive with anti-E2 antibodies (Fig. 7c). Furthermore, ^35^S-amino acid-labelled virus particles at the peak fraction contained several viral proteins, corresponding to core, E1, and E2, all of which are associated with HCV particles (Supplementary Fig. 6A). The putative identities of ^35^S-labelled core and envelope protein E2 were confirmed by immunoprecipitation, followed by autoradiography (Supplementary Fig. 6B). It was notable that the virion contained relatively more envelope proteins than core proteins. Treatment with NP-40 disrupted the virus particles, consistent with its identity as an enveloped virion (Supplementary Fig. 6C). The sum of these analyses confirmed that the viral particles produced from the SB-transfected cells in this system had outer cell surface markers consistent with authentic HCV particles.

To determine whether the viral particles released were infectious, we harvested the culture media from SB RNA-transfected Raji cells (sh-*RIGI*-Raji) which in turn was used to infect naïve Raji cells. The HCV RNA isolated from infected cells was then analysed 8 days later. UV irradiation of virus-containing media prior to infection prevented viral RNA production (Fig. 7d). Also, pretreatment of the transfected cells with IFN-α or -γ, which inhibits viral RNA replication but not viral attachment, lowered HCV RNA levels in a dose-dependent manner (Fig. 7d). Finally, treatment with Telaprevir^62^, an NS3 (viral protease) inhibitor, or 2′-modified nucleosides as inhibitors of HCV replication (2′-C-methy adenosine) also reduced HCV RNA levels in a dose-dependent manner (Fig. 7e).

Active viral RNA replication was also confirmed by the detection of (−) strand RNA in SB-transfected but not JFH1-transfected sh-*RIGI*-Raji cells (Supplementary Fig. 6D). Addition of anti-E2 neutralizing antibody prevented (−) strand RNA synthesis whereas control, non-specific isotype antibody treatment had no effect on HCV replication (Supplementary Fig. 6D, Lower panel). A quantitative estimate of (+) and (−) strand HCV RNA synthesis showed the former was tenfold higher in abundance than (−) genomic RNA. These results clearly demonstrated that active SB HCV RNA replicates in B cells.

By immunofluorescence studies, we also showed that NS3-positive cells could be detected in lymphotropic SB-transfected Raji cells, but not in Huh7.5.1 cells. In contrast, NS3 was detected in hepatotropic JFH-1-transfected Huh7.5.1 cells, but not Raji cells (Fig. 7f). Transfection of replication incompetent strain SB/JFH1 (GND) led to significantly lower levels of NS3 staining at days 7 and 10 post-transfection (Fig. 7g). As a specificity control, SB chimera containing NS5B GND mutations, which cannot replicate in Raji cells (Construct G), did not yield any HCV RNA. These results indicated that the RNA signal was indeed due to viral infection and replication and not due to trapping of viral particles on the outer cell surface. This finding was confirmed by immunoblot (Supplementary Fig. 6E). Taken together, these results demonstrated that HCV SB preferentially infects, replicates and produces infectious virions in sh-*RIGI*-Raji cells, whereas JFH-1 preferentially infects and replicates in Huh7.5.1 cells.

### Persistent HCV infection of PBMC in a humanized mouse model

**Figure 8:**
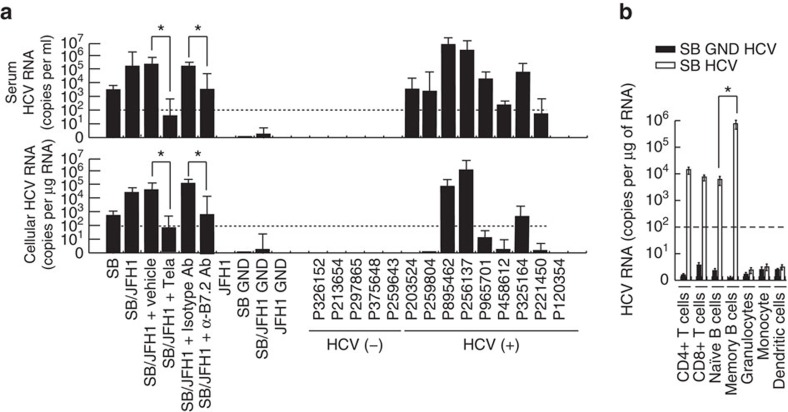
A humanized mouse model engrafted with human hematopoietic cells was utilized to show HCV infection of human lymphomononuclear cells *in vivo* by the lymphotropic HCV strain. (**a**) HCV infection in immunocompromised *Rag2;Il2rg−/−* (RG) mice engrafted with human hematopoietic stem cells (RG-hu HSC Mice) shows that the two HCV strains, SB and JFH-1, have different tissue-specificity. HCV RNA levels in serum and PBMCs isolated from RG-hu HSC mice at 70 days after HCV inoculation were determined by RT-qPCR. Tela: telaprevir (HCV NS3 inhibitor) treatment. (*P<0.05, *t*-test, *n*=3). Error bars represent s.d. (**b**) Human PBMCs from the infected RG-hu mice collected at 70 days after HCV inoculation were separated into various cell types and analysed for the presence of HCV RNA Memory B cells have high cellular HCV RNA levels in comparison to that of naïve B cells. (*P<0.05, *t*-test. *n*=3). Error bars represent s.d.

We extended our study of the lymphotropic properties of HCV-SB *in vivo* by using humanized mice. For this purpose we generated mice with stably engrafted human CD34+ hematopoietic progenitor cells into an immune-compromised background line [*Rag2*−/−*;Il2rg*−/− (RG)-hu HSC mice]. Analysis of these animals (RG-hu HSC mice) following engraftment indicated that 6–37% of immune cells were positive for CD45+ of human origin that was maintained through 10 weeks post-engraftment. After human peripheral blood leukocytes were detected at 16 weeks post-engraftment, the mice were intravenously injected with either cell culture-derived HCV-SB virus, HCV-JFH1 virus or a chimeric SB/JFH1 virus, the latter of which contains the SB 5′-UTR-NS2 genes of the JFH1 genomic backbone (construct F). The HCV RNA levels in the PBMC of these mice were then analysed by RT-PCR at different time points after infection. As shown in Table 1, five of the six mice infected with either SB or SB-JFH1 chimeric virus tested positive for HCV RNA at 4 weeks post-inoculation; four of these six mice remained HCV RNA positive for more than 10 weeks after virus injection. HCV RNA was detected in PBMCs of these mice (up to 2.7 × 10^5^ particles ml^−1^: Fig. 8a). In contrast, mice injected with JFH1 virus or the replication-incompetent strains (GND mutant) of SB or JFH1 strains were negative for HCV RNA.

We infected humanized mice with sera from 14 human subjects, nine of whom tested positive for HCV. None of the HCV-negative sera led to infection in these mice (Table 1). In contrast, 13 of 24 mice inoculated with the HCV-positive sera tested positive for HCV RNA at week 4, of which five mice remained positive up to week 10. Injection of anti-B7.2 antibody reduced both internal and cell surface associated HCV RNA levels (Fig. 8a). Human PBMC from the infected *Rag2*−*/*−*;Il2rg*−*/*− (RG) mice were further sorted into T and B lymphocytes, monocytes, and dendritic cells and examined separately for cellular HCV RNA. As shown in Fig. 8b, the HCV RNA was preferentially detected in memory B cells. HCV RNA was also detected in CD4+ and CD8+ T cells in humanized mice (Fig. 8b). These results indicated that HCV was capable of persistently infecting human B cells in a humanized mouse model.

### HCV binding downregulates B7.2 and inhibits Ig production

**Figure 9:**
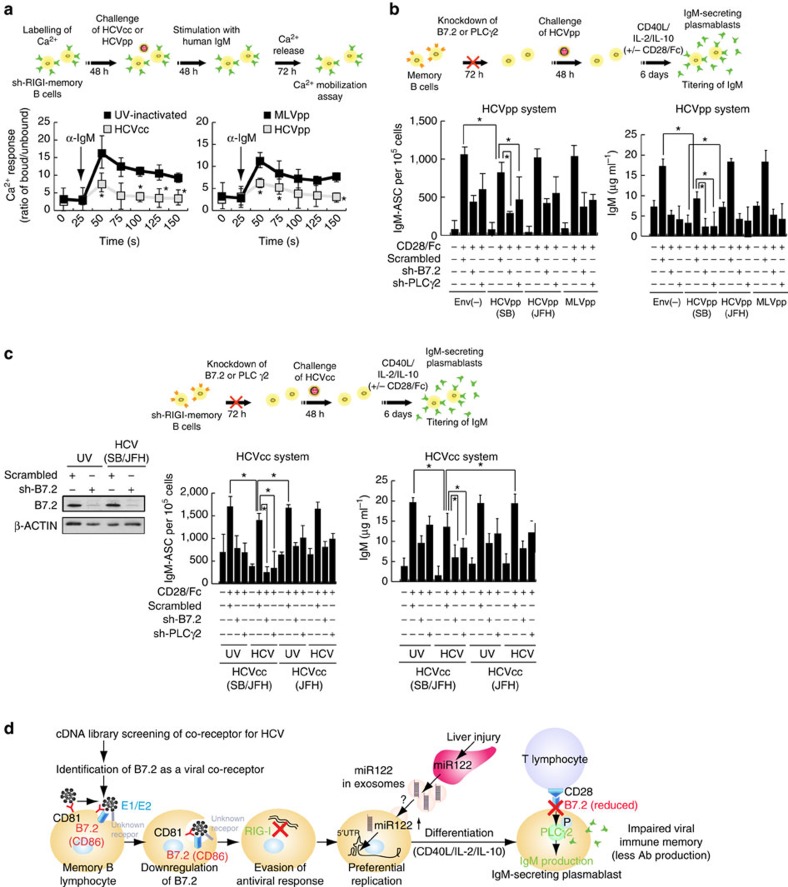
HCV infection impairs antigen recall responses in memory B cell. (**a**) HCV-infected CD27+ B cells, compared to uninfected CD27+ B cells, have attenuated Ca^2+^ responses after BCR cross-linking. B cells from healthy donor were loaded with Indo-1-AM, stained with anti-CD27 and warmed to 37 °C. After establishing a baseline for 30 s, cells were stimulated with 10 μg ml^−1^ goat F(ab′)2 anti-human IgM. Kinetic graphs represent ratios of bound/unbound Indo-1 AM over time for CD27+ B-cell populations. Arrows indicate addition time of F(ab′)2α–human IgM (*P<0.05, *t*-test, *n*=3). Error bars represent s.d. (**b**) Signals by anti-B7.2 Ab induce the production of IgM by memory B cells. Memory B cells (5 × 10^3^ cells per well) were cultured with different concentrations of anti-B7.2 (0.5 μg ml^−1^); after 5 days, supernatants from control and experimental wells were collected and IgM levels were measured. Data are presented as the fold-increase above memory B cells activated with a CD40L+IL-2+IL-10, and a species- and isotype-matched control Ab (*P<0.05, *t*-test, *n*=3). Error bars represent s.d. (**c**) Similar assays were performed using HCVcc as described with HCVpp above (*P<0.05, *t*-test, *n*=3). Error bars represent s.d. (**c**, *inset*) Silencing effect of shRNA targeting B7.2 was confirmed by immunoblot. (**d**) A model of lymphotropic HCV infection in memory B cells. B7.2 serves as one of the co-receptors for HCV-SB infection of B cells. B7.2 (CD86) mediates lymphotropism of HCV infectious clone derived from HCV SB strain towards human memory B cells and its binding suppresses the cells' function by downregulating surface expression of B7.2. Evasion of antiviral responses (that is, RIGI), induction of *miR-122*, and another host factor targeting HCV 5′-UTR are involved in stimulating HCV replication in B cells. HCV-mediated downregulation of B7.2 impairs anti-viral immune memory, including reduction of antibody production and antigen recall responses.

As CD27+ memory B cells in HCV patients are anergic (exhausted) B cells^63^,^64^, the functions of memory B cells infected with HCV particles were examined accordingly. To determine if HCV-infected CD27+ B cells had attenuated signalling upon BCR ligation, Ca^2+^ mobilization was measured after immunoglobulin (Ig)M stimulation with anti-human IgM. The HCV-infected CD27+ B cells had diminished Ca^2+^ mobilization (Fig. 9a), indicating that HCV-infected CD27+ B cells are anergic to BCR-mediated stimulation. To test if HCV-infected cells were prone to apoptosis, HCV-infected primary memory B cells isolated from patients were stimulated with CD40L+IL2+IL-10 or vehicle control *ex vivo* (Supplementary Fig. 9A). Treated memory B cells were stained with annexin V (to measure apoptosis) and anti-CD27. After incubation with vehicle control or CD40L+IL2+IL-10 treatment, the percentage of annexin V^+^ cells was higher in the CD40L+IL4-stimulated HCV-infected B-cell subset than that of UV-irradiated supernatant-treated cells (Supplementary Fig. 9A). As CD40L stimulation transactivates pro-apoptotic molecule *Bax* (ref. 65), *Bax* mRNA levels were also examined. The treatment was found to induce BAX after cross-linking B7.2 on HCV-infected memory B cells (Supplementary Fig. 9A, right). These data suggested that, in the absence of survival signals, HCV-infected CD27+B cells were prone to apoptosis.

B7.2 is a costimulatory molecule that interacts with CD28 and CTLA-4 on T cells. In addition, B7.2 has a cytoplasmic tail that transduces signals in antigen-presenting cells^66^,^67^. B7.2 stimulation results in the phosphorylation of phospholipase Cγ2 (PLCγ2) and protein kinase C αβ (PKCα/β), leading to activation of transcription factor Oct-2 and eventual increased IgG1 transcription and protein production^68^. Since binding of the HCV E2 protein to CD81 can perturb the biological activities of CD81 (ref. 69), we tested if the binding of HCV E2 to B7.2 also affected B7.2 and its signalling pathways. To determine whether HCVpp-SB infection reduced CD28/Fc-mediated activation of PLCγ2 and PKCα/β, we analysed the activation of PLCγ2 and PKCα/β. As shown in Supplementary Fig. 9B, CD28/Fc-mediated phosphorylation of PLCγ2 (Tyr^1217^) and PKCα/β (Thr^638/641^) was reduced in HCVpp-infected memory B cells.

To test if the observed effects on signalling were due to the E2–B7.2 interaction, we used purified recombinant E2 (SB strain) purified from Baculovirus expression system instead of HCVpp-SB. To test if E2 (SB strain) binds to B7.2, His-tagged E2 (without the transmembrane domain) from SB strain (genotype 2b) and H77 strain (genotype 1a) were expressed in the baculovirus expression system and partially purified (Supplementary Fig. 7A). The intracellular E2 was detected in both monomeric and aggregated forms (Supplementary Fig. 7B). Immunoblot analysis confirmed expression of HCV E2 protein (Supplementary Fig. 7C). The partially purified E2 was used for *in vitro* binding to various cell lines as determined by flow cytometry. The results showed that E2 from the SB strain bound to Raji cells very efficiently. E2 from the genotype 2a JFH1 strain does not bind to HepG2 cells (Supplementary Fig. 7D). The binding strengths of E2 from SB and JFH1 strains were further compared by using different amounts of E2 (normalized using an anti-His_6_ antibody). E2 did not bind to HepG2 cells, which is among the few human cell lines that do not express CD81 or B7.2. In contrast, E2 bound to the B7.2-overexpressing HepG2 cells efficiently (Supplementary Fig. 7D). The binding was partially blocked by a B7.2-specific antibody but not by an isotype-matched control antibody (Supplementary Fig. 7D). As shown in Supplementary Fig. 7E, E2 of the SB strain bound more than that of the H77 strain at every protein concentration used. These results indicated that recombinant E2 (SB lymphotropic strain), but not E2 (H77 hepatotropic strain), bound to B cells in a B7.2-dependent manner.

Furthermore, to rule out that the observed effects were not a consequence of CD81 engagement by E2-SB, endogenous CD81 was knocked out by CRISPR and replaced with exogenous full-length CD81 or extracellular-domain-truncated CD81 expression vectors. CD81 overexpression partially restored immunoglobulin production in CRISPR-mediated CD81 knockout cells (Supplementary Fig. 8A). To further test if another co-receptor SR-BI stimulated immunoglobulin production, SR-BI was knocked out by CRISPR and replaced with transfected full-length SR-BI or intracellular-domain-truncated mutant of SR-BI and examined for immunoglobulin production. Both C-terminus truncation mutant and full-length SR-BI showed no effect on IgM production in the CRISPR SR-BI knockout cells expressing C-terminus truncation mutant of SR-BI or full-length SR-BI (Supplementary Fig. 8B). B2.7 restoration promoted IgM production while B7.2/CD8 chimera, which lacks the signal transduction domain, failed to restore the IgM production (Supplementary Fig. 8C, left). These results indicated that HCV E2(SB)-mediated downregulation of B7.2 reduced CD28/Fc-mediated signalling pathways.

### HCV-infected B cells do not differentiate into plasmablasts

To test whether binding of HCV to B7.2 inhibited B7.2-induced signalling pathways, resting B cells were purified from human PBMC for challenge with HCVpp-SB. As B7.2 signalling promotes the IgM production, supernatants were collected on day 6 following the administration of CD40L+IL-2+IL-10 and assayed by enzyme-linked immunosorbent assay (IgM ELISPOT assay) for IgM production from these cells. Memory B cells cultured with medium alone or with control antibody did not induce antibody secretion. As illustrated in Fig. 9b, memory CD27^+^ B cells were infected with HCVpp and stimulated with CD40L, IL-2 and IL-10 for 6 days, which differentiated B cells into antibody-secreting cells. Similarly, treatment with CD28/Fc fusion protein induces B7.2 signalling and activates Ig production pathways^68^. The pseudotyped MLV viral particle, which does not bind to B7.2 but infects B cells, did not affect CD28-mediated IgM production (Fig. 9b). We observed that the HCVpp-infected CD27^+^ B cells were less efficient at differentiating into IgM-secreting plasmablasts following CD40L/IL-2/IL-10 stimulation (Fig. 9b, left). This suggested differentiation to IgM-secreting plasmablasts upon CD40L/IL-2/IL-10 stimulation was impaired in HCVpp-infected memory B cells, compared to uninfected memory B-cells (Fig. 9b, left). These results indicated that HCVpp-SB-mediated downregulation of B7.2 reduced CD28/Fc-mediated signalling pathways. Silencing B7.2 or PLCγ2 significantly reduced IgM production in HCV-infected memory B cells (Fig. 9b, right). In contrast, HCVpp-JFH1, which does not bind to B7.2, did not inhibit IgM production. To confirm the biological relevance of the pseudotyped HCV particle study, similar results were also obtained when we used the HCV-SB virus (Fig. 9c). Class switch did not account for the decreased IgM in these subsets, as we consistently detected<140 ng ml^−1^ of IgM in cell culture supernatants. Taken together, these results indicated that infection with both HCVcc and HCVpp increased IgM production and by downregulation of B7.2, attenuated CD28-mediated IgM production; thus virus infection enhanced the differentiation of memory B cells into IgM-secreting plasmablasts.

## Discussion

In this study, we have established the genetic basis for lymphotropism of HCV infection. A comprehensive set of data indicated that B7.2 is a co-receptor for observed HCV SB strain tropism towards memory B cells. Previous reports have also indicated that some HCV strains can infect human T cells: the H77 strain of HCV can infect and replicate in established T-cell lines, such as Molt-4 and Jurkat cells, in long-term culture^21^. Furthermore, transfection of SB strain RNA, but not JFH1, led to its replication in Molt-4 cells^70^. Here we provide evidence indicating that B7.2 is involved in HCV SB strain tropism towards memory B cells (additional discussion is described in Supplementary Note 1).

B7.2 belongs to the immunoglobulin superfamily, and is highly expressed on memory B cells and germinal centre B cells^71^. Its ligand includes CD28 and CTLA4 on the surface of T cells. B7.2 and B7.1 are expressed by antigen-presenting cells including B cells. The result of ligation of B7.2 leads to the following: B7.2-CD28 interaction co-stimulates T-cell activation and enhances T-cell survival, B7.2-CTLA4 interaction inhibits T-cell activation and leads to T-cell tolerance. The infection of B cells by HCV likely causes significant alteration of these functions^72^. Furthermore, expression of HCV viral proteins in B cells can result in B-cell lymphoma in transgenic mice^73^. The expression levels of B7.2 are not correlated with cellular HCV RNA levels in certain primary cells, such as dendritic cells—which are known to be associated with HCV^74^—indicating that other co-receptor(s) may exist for lymphocyte infection (Supplementary Fig. 5G). Indeed, CD5 expression is linked to HCV infection of lymphocytes^75^. Another possibility is that inhibitors of HCV infection, such as Ewi-2wint, a partner of CD81, may be associated with this lymphotropism^76^. Other studies have shown ApoE to be a tropism determinant in a late assembly step and viral cell-to-cell transmission of HCV JFH1 strain^77^.

In conclusion, HCV tissue tropism is determined primarily by the properties of viral envelope proteins and the 5′-UTR, in addition to the ability of HCV to evade antiviral responses (Fig. 7f). The existence of lymphotropic HCV strains indicates that lymphoid cell infection may be an important facet of HCV infection in some patients. These studies on the genetic basis of lymphotropism of HCV will open up an avenue for studying extrahepatic infection of HCV *in vivo* and *in vitro*. The SB strain may be representative of lymphotropic quasispecies that arise from an HCV infection that is primarily hepatotropic.

## Methods

### Cells

Raji (Cat# CCL-86), HEK293T (Cat# CRL-3216) and HepG2 (Cat# HB-8065) cells were obtained from ATCC. HepG2 cells were transfected with pCDNA3.1-CD81 or B7.2 expression plasmid, selected with G418 (0.5 μg ml^−1^) and surviving cell colonies were picked after drug selection. Individual cell clones were further isolated and characterized for CD81 expression and E2 binding. To generate sh-*RIGI*-Raji cells, Raji cells were transduced by multiple lentivirus expressing shRNAs against RIG-I and MDA5 (Open Biosystems) and/or miR122. The culture media for Raji and sh-*RIGI*-Raji cells is RPMI media 1640, GlutaMAX (Cat#61870-036, Life Technologies) containing 20% FBS (Omega, Inc.). PBMC were isolated by Ficoll-Paque Plus (Amersham Biosciences) density gradient centrifugation. Informed consent was obtained. Institutional Review Board at University of Southern California approved the procedures. PBMC were maintained in RPMI 1640 containing 10% FBS. Infection of cells, generation of HCV pseudo-particles and generation of HCV clone ar described in Supplementary Methods.

### Vector

The Claudin-1 expression vector was a gift from Dr Weeraratna from National Institute of Aging (NIA/NIH). WT B7.2, B7.2-CD8, ICAM-1/CD8 and WT ICAM-1 expression vectors were previously described (gifts from Dr Jung, USC) (ref. 78).

### Sucrose density gradient analysis

The viral particles produced from the transfected cells were concentrated from the culture supernatant and separated by sucrose gradient sedimentation. Culture medium derived from sh-*RIGI*-Raji cells was harvested for sucrose density gradient analysis 6 days after transfection of full-length SB RNA. Collected culture medium was cleared by low speed centrifugation at 670*g* for 10 min, and passed through a filter with 0.45-μm pore size (Millipore). Filtered culture medium was layered on a stepwise sucrose gradient (60 to 10%, wt/vol) and centrifuged for 16 h in a SW41 rotor (Beckman) at 40,000 or 50,000 rpm (273,865*g* or 427,914*g*) at 4 °C. After centrifugation, 8 or 16 fractions were harvested from the bottoms of the tubes. Core protein concentration in each fraction was determined by an immunoassay using 100 μl of the fraction. HCV RNA titer was determined by RTD-PCR using RNA isolated from 100 μl of the fraction. The ^35^S labelling was performed by standard procedures.

### Lentivirus production

To generate *RIGI* or *MDA5* shRNA or a *miR-122* lentivirus, the transfer vector expressing *miR-122* or shRNA targets sequence on the RIG-I or MDA5 cDNA (RHS4531-NM_022168, RHS4430-98851910, RHS4430-99158156, RHS4430-99161339*: MDA5* so called *DDX58, or* RHS4531-NM_014314, RHS4430-98910525, RHS4430-99294017, RHS4430-99619590: RIG-I so called DDX58; Human GIPZ Lentiviral shRNAmir target gene set, Open Biosystems) were co-transfected with pPAX2 and pVSV-Gp expression vector in HEK293T cells.

### Cell entry assay

Virus infection was carried out at 4 °C (no endocytosis) and then shifted to 37 °C (active endocytosis). Antibody-treated and untreated cultures were infected with HCVpp(SB) or HCVpp(JFH1) for 1 h at 4 °C. Plates were then brought to 37 °C and assayed at indicated time points over a 300 min time course. Controls are normalized to the value for anti-CD81 added at 4 h. Antibodies directed against CD81 (JS81) or B7.2 were added at various time points after temperature was shifted to 37 °C, and the infectivity was determined at 12 days after infection as previously described. The concentration of B7.2 and CD81 we used in the experiments are both 50 μg ml^−1^. Half-maximal inhibition time of anti-B7.2 was 132 min while half-maximal inhibition time of anti-CD81 was 44 min.

### Immunoprecipitation followed by immunoblot for B7.2

Full-length of B7.2, B7.2/CD8, full-length of ICAM or ICAM/CD8 were overexpressed in 293T cells. After 24 h, the expression level of B7.2 was determined by western blots. For infection, the culture media was replaced with fresh media containing either SB HCVpp or JFH1 HCVpp and incubated an additional 24 h. Cell lysates were used for mmunoprecipitation with anti-B7.2, followed by immunoblot for B7.2 proteins in 293T cells.

After incubation, the cells were harvested and lysed for immunoprecipitation. B7.2 only interacted with HCV E2 after SB infection, but not JFH1 infection. These data indicate that SB E2 protein, but not JFH1 E2 protein, directly interacted with B7.2.

### Electron microscopy

Raji cells were transfected with HCV RNA and cell-free supernatant was harvested 96 h post transfection and concentrated via ultracentrifugation. Grids were washed on drops of PBS, fixed with 3% paraformaldehyde in PBS and blocked in a solution of 0.8% BSA, 1% cold water fish skin gelatin (Sigma) and 20 mM Glycine in PBS. Immunogold labelling was performed with an antibody directed against E2 (CBH5) diluted 1:50 in blocking solution, and Protein A coupled to 10 nm gold particles. After extensive washing with PBS and a quick rinse with distilled water, grids were stained with a solution consisting of 9 parts 2% Methyl Cellulose and 1 part 3% Uranyl acetate (both aqueous solutions).

### Isolation of human B cell

To isolate human memory and naïve B cells from 14 healthy individuals and 18 HCV patients, EasySep Human Memory B Cell Isolation Kit and EasySep Human Naïve B Cell Enrichment Kit (STEMCELL Technologies, Co. Ltd, USA) were used, respectively according to instruction manuals. IRB approval is obtained by University committee. Exosome isolation is described in Supplementary Methods.

### Immunoblot

Proteins were detected by designated antibodies as following: MDA5 (D74E4, Rabbit mAb, Cell Signaling #5321S, 1:1,000), MDA5 (Santa Cruz, sc-48031, 1:1,000), RIG-I rabbit Ab (Cell Signaling, #4520S, 1:1,000), RIG-I (Santa Cruz, sc-48929, 1:1,000), B7-2 (BU63, sc-19617, Santa Cruz Biotechnology, 1:1,000), B7.2 (Santa Cruz, sc-28347, 1:1,000), Claudin-1 mAb (ZYMED, #37-4900, 1:2,000). HCV E2 was detected by CBH5 or CBH17 antibodies (kind gifts from Prof. Steven Foung in Stanford University, Palo Alto, CA, USA, 1:1,000). The phosphorylation of PLCγ2 (Tyr^1217^: Cell Signaling cat#: 3871 T, 1:2,000) and PKCα/β II (Thr^638/641^: Cell Signaling cat#: 9375 T, 1:2,000) were analysed using antibodies directed against their respective phosphorylated forms. Total protein levels were determined using antibodies not specific for the phosphorylated forms of these proteins. Uncropped scans of western blots are provided in Supplementary Fig. 10.

### Co-receptor cloning by lentivirus B-cell cDNA library screen

To identify the possible novel receptor specific for the infection of lymphotropic HCV, we performed a lentivirus-based screen of a cDNA library, derived from the highly HCV-permissive B lymphoma Raji cell lines, for genes that render the non-permissive CD81^+^ SR-BI^+^ HEK293T cell line infectable with HIV-1 particles pseudo-typed with HCVgp (HCVpp). More details are described in Supplementary Methods.

### RNA synthesis

XbaI-digested constructs were treated with mung bean nuclease (New England Biolabs) to remove 4 nucleotides and leave the correct 3′ end of the HCV cDNA. Digested plasmid DNAs were purified and used as templates for RNA synthesis. The HCV RNA was synthesized *in vitro* using the MEGAscript T7 kit (Ambion, Austin, TX, USA). Synthesized RNA was treated with DNaseI (RQ1 RNase-free DNase, Promega) followed by acid phenol extraction to remove any remaining template DNA.

### Baculovirus expression and purification of HCV E2 proteins

E2 sequences from a genotype 2b isolate (strain SB) and genotype 2a isolate (strain JFH1) without the C-terminal transmembrane domains but containing a His6 tag at the C termini were cloned into a transfer vector (pBlueBacHis2; Invitrogen, Carlsbad, CA, USA). Expression of recombinant E2 proteins in insect cells (Sf9) was performed as described in the Bac-N-Blue Baculovirus Expression System (Invitrogen) and purified as previously described^79^. Briefly, insect Sf9 cells were grown in Grace's Insect medium supplemented with 10% FBS at 28 °C. The bacmid DNAs were transfected into Sf9 cells; the virus generated was amplified two more times at a low multiplicity of infection before being used for infection of Sf9 cells (at high multiplicity of infection). At 72 h post-infection, cells were lysed in 50 mM Tris-HCl (pH 8.5), 0.15 M NaCl, 0.1% Triton X-100 and protease inhibitor cocktail (Roche, Basel, Switzerland) and then sonicated and centrifuged at 100,000*g*. E2 were purified from the crude lysates by using HiTrap chelating columns (Amersham Biosciences, Piscataway, NJ, USA) loaded with 0.1 M NiCl2 (ref. 80). The extracts were diluted in 50 mM sodium phosphate (pH 8), 300 mM NaCl and 10% glycerol (buffer A) and bound to the column (flow rate: 0.5 ml min^−1^). The column was washed sequentially with buffer A, buffer B (50 mM sodium phosphate [pH 6], 300 mM NaCl, 10% glycerol) and buffer C (50 mM sodium phosphate [pH 8], 1 M NaCl, 10% glycerol). The proteins were eluted in buffer A containing 200 mM imidazole and buffer exchanged into phosphate-buffered saline without Ca2+ and Mg2+[PBS(−)] plus 10% glycerol.

### Statistical analysis

ANOVA analysis was used for multiple comparisons. For correlation analysis, Pearson's correlation test was performed in GraphPad Prism software. For all other statistical analyses, the nonparametric Mann-Whitney test or two-tailed *t*-test was employed. Values of *P*<0.05 were considered to be statistically significant.

### Data availability

The sequence of the HCV SB clone has been deposited in the GenBank Nucleotide database with the accession code KM349851. The authors declare that all other relevant data are available from the authors on request.

## Additional information

**How to cite this article:** Chen, C.-L. *et al*. Hepatitis C virus has a genetically determined lymphotropism through co-receptor B7.2. *Nat. Commun.* **8,** 13882 doi: 10.1038/ncomms13882 (2017).

**Publisher's note:** Springer Nature remains neutral with regard to jurisdictional claims in published maps and institutional affiliations.

## Supplementary Material

Supplementary Information Supplementary Figures, Supplementary Notes, Supplementary Methods and Supplementary References

## Figures and Tables

**Table 1:** HCV infection analysis on immunocompromised mice transplanted with human hematopoietic stem cells (HSCs).

Viral inoculum*	**Humanized mice inoculated (*n*)**	**HCV(+) mice at week 4**†	**HCV(+) mice at week 10**†	**HLA positivity**‡
*Culture supernatant*				
SB	3	2	2	+(26)
SB/JFH1	3	3	2	+(31)
SB/JFH1+ Vehicle	3	3	3	+(27)
SB/JFH1+Telaprevir	3	1	1	+(29)
SB/JFH1+Isotype Ab	3	3	3	+(26)
SB/JFH1+ a-B7.2	3	1	1	+(32)
JFH1	3	0	0	+(34)
SB GND	3	0	0	+(33)
SB/JFH1	3	0	0	+(35)
JFH1 GND	3	0	0	+(29)
				
*Human subjects*				
P326152	3	0	0	+(28)
P213654	3	0	0	+(18)
P297865	3	0	0	+(33)
P375648	3	0	0	+(14)
P259643	3	0	0	+(17)
P203524	3	2	0	+(33)
P259804	3	2	0	+(15)
P895462	2	2	2	+(14)
P256137	3	1	2	+(36)
P965701	3	2	0	+(37)
P458612	3	2	0	+(6)
P325164	3	2	1	+(19)
P221450	3	0	0	+(18)
P120354	3	0	0	+(28)

^*^Viral inocula were obtained from nine different chronic HCV patients (coded) or from cell culture-derived HCV (that is, SB, SB/JFH1 chimera and JFH1).

^†^HCV positivity was determined by RT-PCR for HCV positive-strand RNA.

^‡^HLA DNA was determined by FACS using antibody that is specific to human HLA.
