# Supplementary material for: Hepatitis C virus has a genetically determined lymphotropism through co-receptor B7.2
Source: Nat Commun. 2017 Jan 9;8:13882. doi: 10.1038/ncomms13882 (PMC5227552; doi:10.1038/ncomms13882)
Supplement: Supplementary Information — Supplementary Figures, Supplementary Notes, Supplementary Methods and Supplementary References [file ncomms13882-s1.pdf]

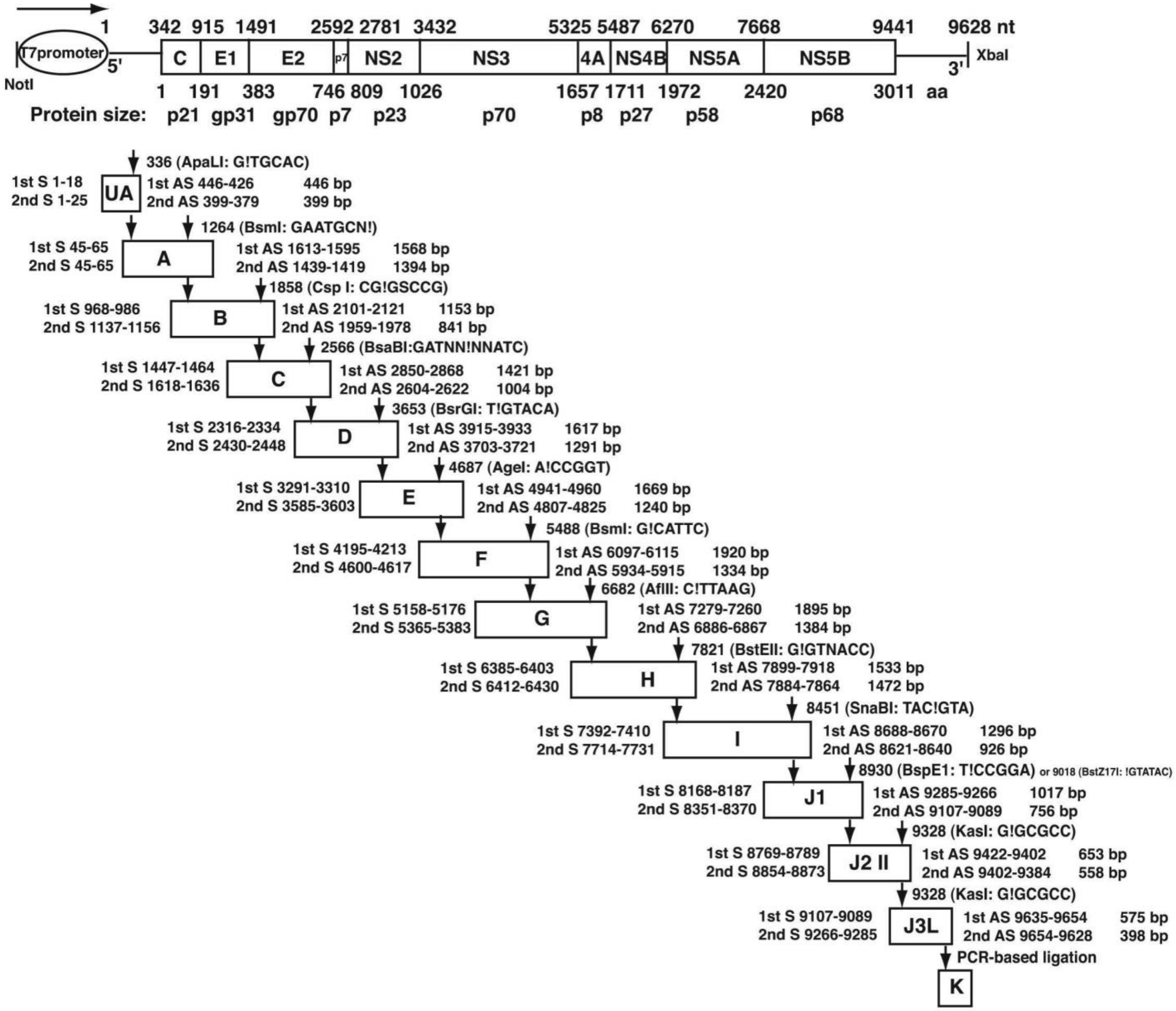

Supplementary Figure 1. Assembly of PCR fragments of HCV SB strains.
The nucleotide numbers are shown for the first and second primer sets to amplify the PCR fragments. Nucleotide length of each PCR fragment is shown. Arrows indicate the cleavage sites of restriction enzymes.

Supplementary Figure 1
Chen et al.

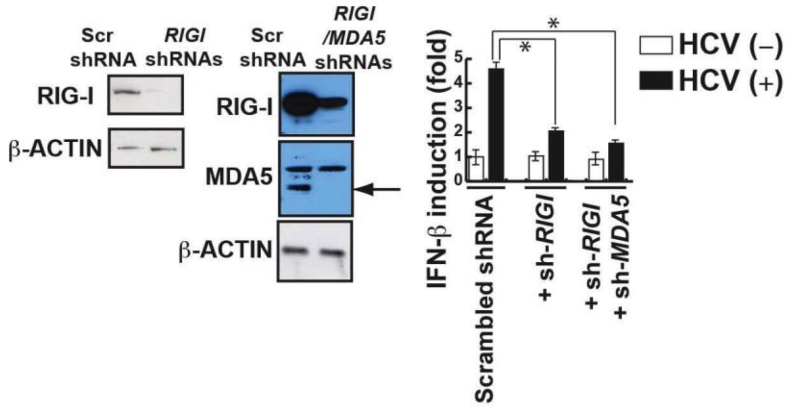

Supplementary Fig. 2. Immunoblot analysis of RIGI, MDA5, and β-ACTIN, and qRT-PCR of IFN-β production after treatment of Raji cells with scrambled shRNA, RIGI or MDA5 shRNA (n=3). *P<0.05, by Student's *t*-test. Error bars represent standard deviation.

Supplementary Figure 2
Chen et al.

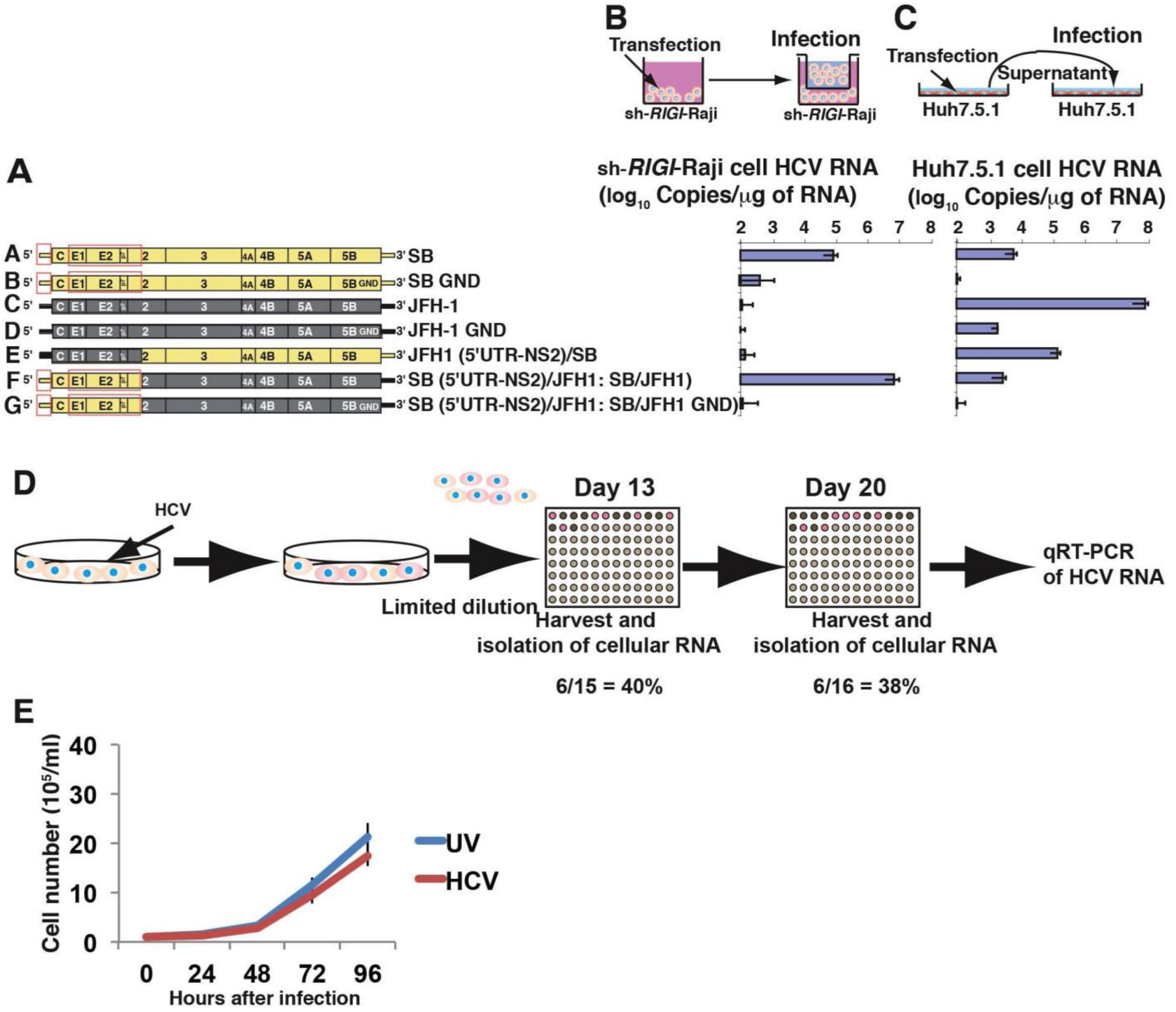

Supplementary Fig. 3. Supernatant of HCV transfected cells contain infectious virus. (**A**) The constructs used for determination of HCV RNA titers. (**A** and **B**) The passage of virus in the Raji (sh-RIGI-Raji) culture supernatant. The supernatant harvested from the HCV RNA-transfected cells at day 24 was used to infect Raji (sh-RIGI-Raji). Error bars represent standard deviation (*n* = 3). (**B**) or Huh7.5.1 cells (**C**). (**D**) Infectivity was directly quantified by limited dilution assay followed by HCV qRT-PCR of intracellular RNA. (**E**) Cell growth curve of HCV-infected Raji cells as determined by Trypan blue exclusion assay.

Supplementary Figure 3
Chen et al.

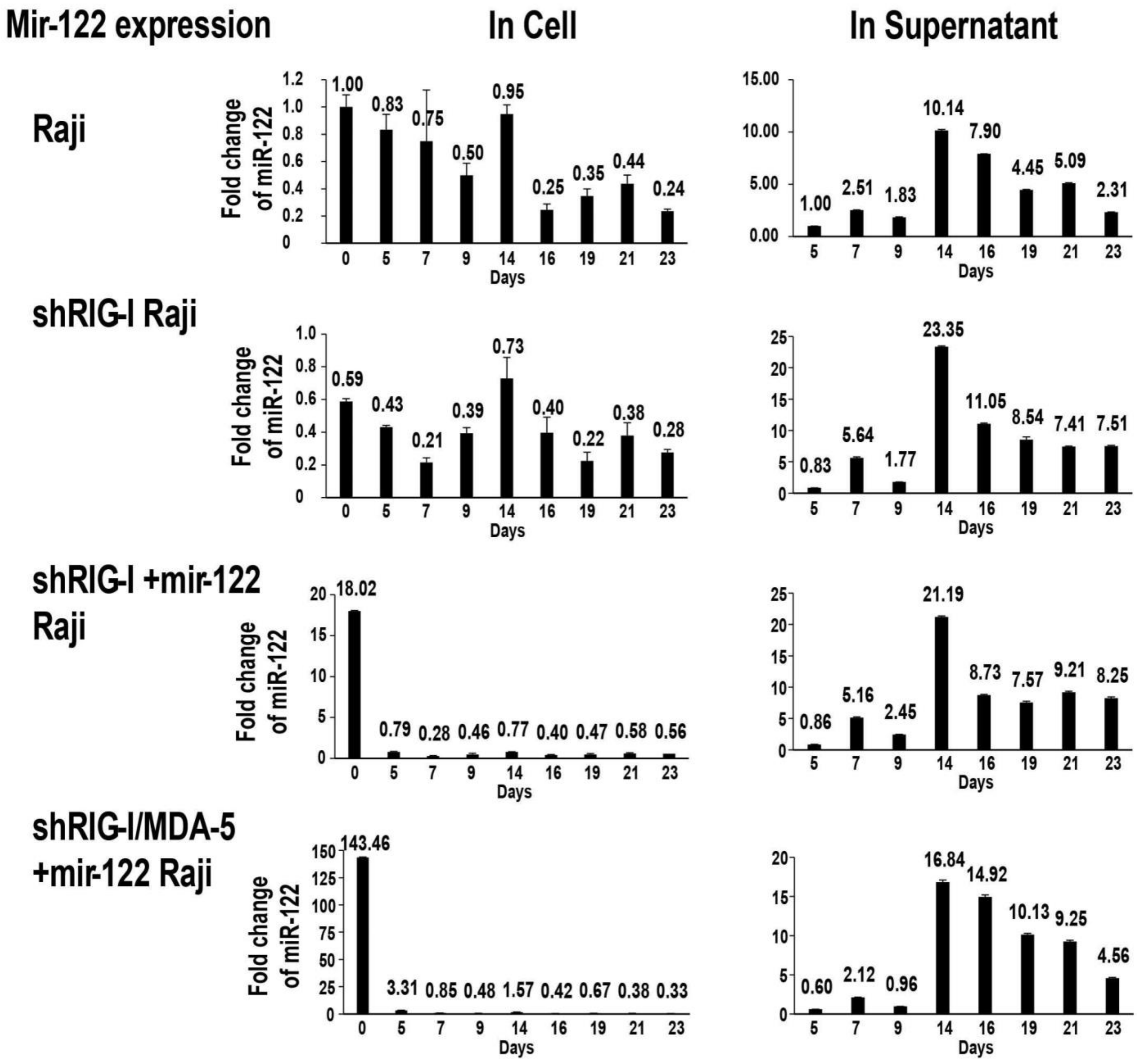

Supplementary Fig. 4. miR-122 is secreted from HCV-infected B cells. Cellular miR-122 levels were significantly reduced four days post-transfection while levels of miR-122 significantly increased in supernatant Indeed, HCV RNA levels in supernatant significantly increased at day 7 and further increased at day 14 post-transfection. Error bars represent standard deviation (*n* = 3).

Supplementary Figure 4
Chen et al.

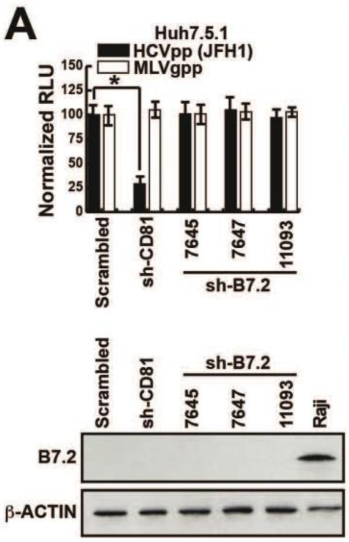

B

Expression Cloning of Lymphotropic HCV Receptor Identifies Candidate Genes

Name	Full description	Feature	Localization
B7.2	CD86	T cell survival/tolerance	Cell surface
ATP2B1	ATPase, Ca^{2+} transporting, plasmamembrane 1	Removal of Ca^{2+} from cells	Plasma membrane
TRIM66	Tripartite motif-containing 66	Abundant in spleen, but no expression in liver Zinc ion binding, Heterodimerization with CBX1	Transcription repressor
CAP1	CAP, adenylate cyclase-associated protein 1	Cyclic AMP pathway, Filament dynamics, Actin binding	Cytoplasm
EEF1A1	Eukaryotic Translation Elongation Factor 1 a1	Key lipid metabolism enzyme: activate SREBP1	Cytoplasm
ZNF580	Zinc Finger Protein 580	LDL-induced EC protein, Zinc ion binding	Nuclei
ZNF581	Zinc Finger Protein 581	LDL-induced EC protein, Zinc ion binding	Nuclei
MBTPS1	Membrane-bound Transcription Factor Peptidase	Transcription factor protease	Membrane
RPL3	Ribosomal Protein L3	Ribosomal protein	ER
REX4	RNA exonuclease 4 homolog	RNA endonuclease	Nucleus

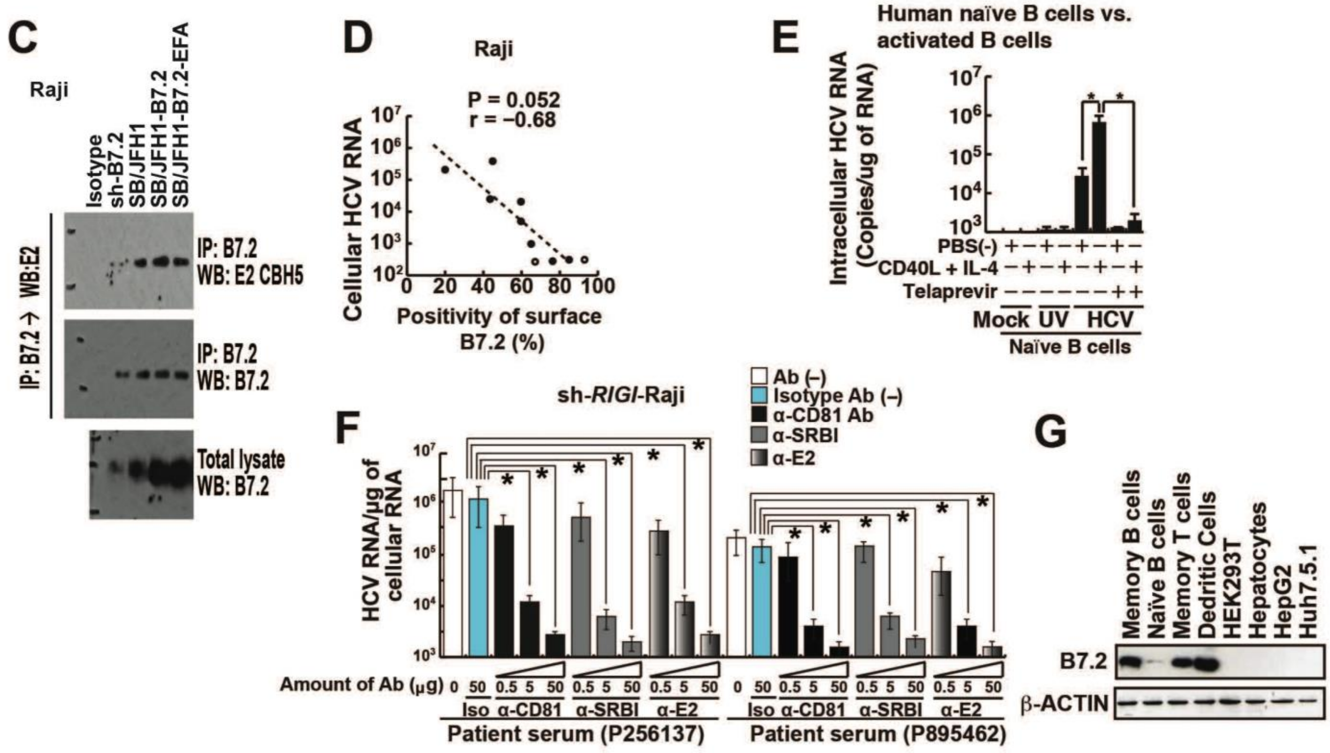

Supplementary Fig. 5. Memory B cells are infected with HCV with downregulation of B7.2. (**A**) B7.2 silencing does not inhibit HCVpp(JFH1) entry into Huh7.5.1 cells. (**bottom**) Immunoblot of B7.2 is shown in Huh7.5.1 cells. Error bars represent standard deviation (*n* = 3). (**B**) Several candidate receptors were cloned from selected HEK293T cells with B cell cDNA library. (**C**) Immunoprecipitation in B7.2-silencing Raji cells. Full-length of B7.2 or B7.2/CD8 was overexpressed in B7.2-silencing Raji cells. Cells were then electroporated with SB/JFH1 virus. After 24 hours, cells were harvested and lysed for immunoprecipitation in reciprocal way. As shown in figure, SB E2 directly interacted with B7.2. However, this data cannot conclude that SB E2 directly interacted with B7.2 at viral entry step. (**D**) Correlation plot between B7.2 levels and relative infectivity. (**E**) Detection of HCV RNA in human naïve B cells vs. activated B cells treated with CD40L and IL-4. Error bars represent standard deviation (*n* = 3). (**F**) The blocking effects of anti-CD81, anti-SRBI and anti-E2 on HCVpp(SB) infection. Error bars represent standard deviation (*n* = 3). (**G**) Immunoblot analysis of B7.2 expression in different cell types. *$P<0.05$, by Student's *t*-test.

Supplementary Figure 5
Chen et al.

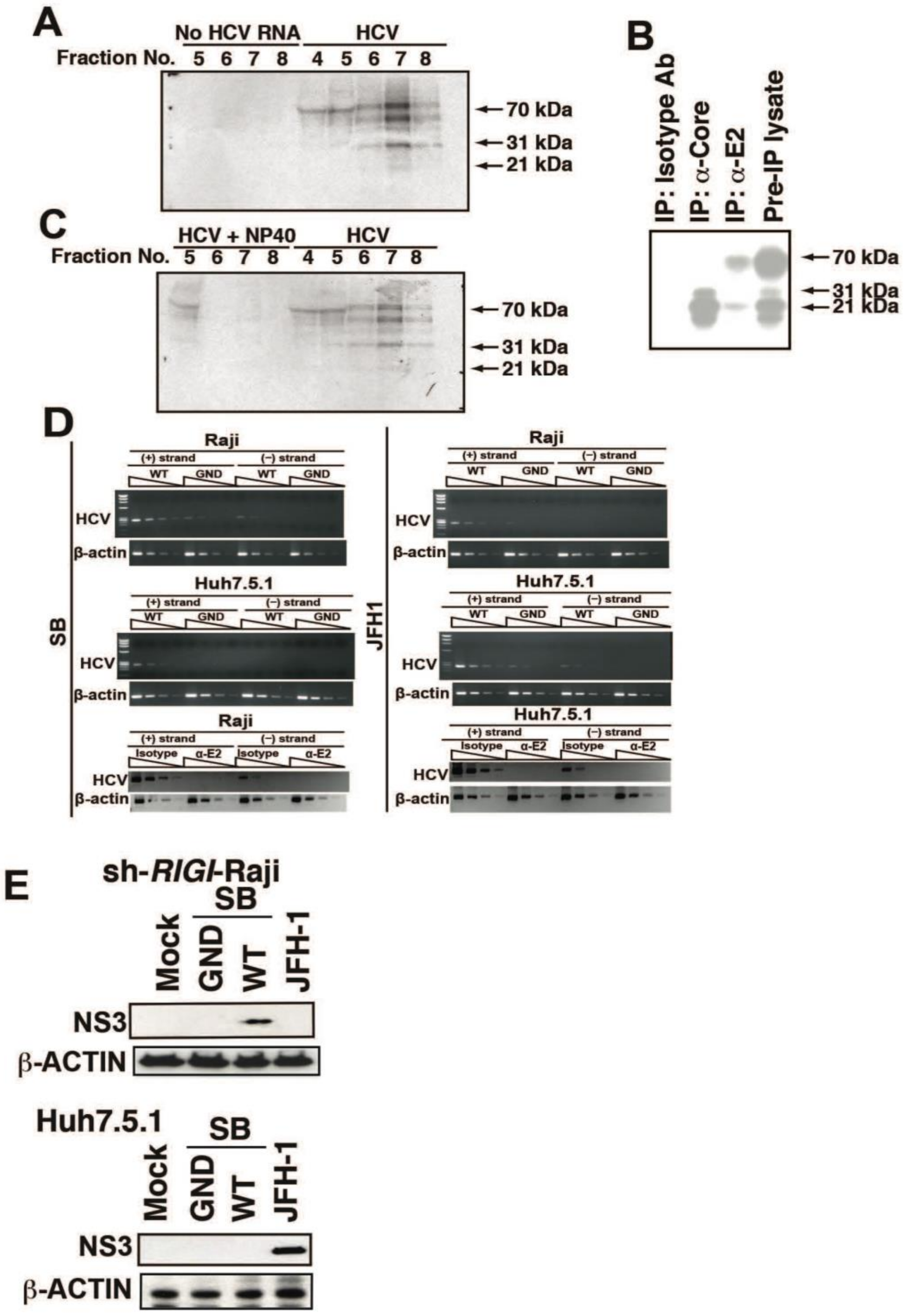

Supplementary Fig. 6. Detection of viral proteins in HCV particles using sucrose gradient centrifugation, semi-quantitative RT-PCR and metabolic labeling. (**A**) Density gradient fractions of [^{35}S] amino acid labeled cell culture supernatant. Each fraction was separated by SDS-polyacrylamide gel electrophoresis. (**B**) Immunoprecipitation of Core or E2 proteins using antibodies in HCV-RNA-transfected cells. Isotype-matched antibody was used as negative control. (**C**) Density gradient fractions of [^{35}S] amino acid labeled cell culture supernatant. Same as in (**A**), except that the same viruses were treated with NP-40 before sucrose gradient sedimentation. (**D**) Detection of negative-strand RNA of SB HCV in Raji, but not in Huh7.5.1 cells. Strand-specific semi-quantitative RT-PCR was performed to detect (+)- and (−)-strand RNA. (**E**) RIG-I silenced cells are infected with SB or JFH-1 HCV. Expression of NS3 is confirmed by immunoblot.

Supplementary Figure 6
Chen et al.

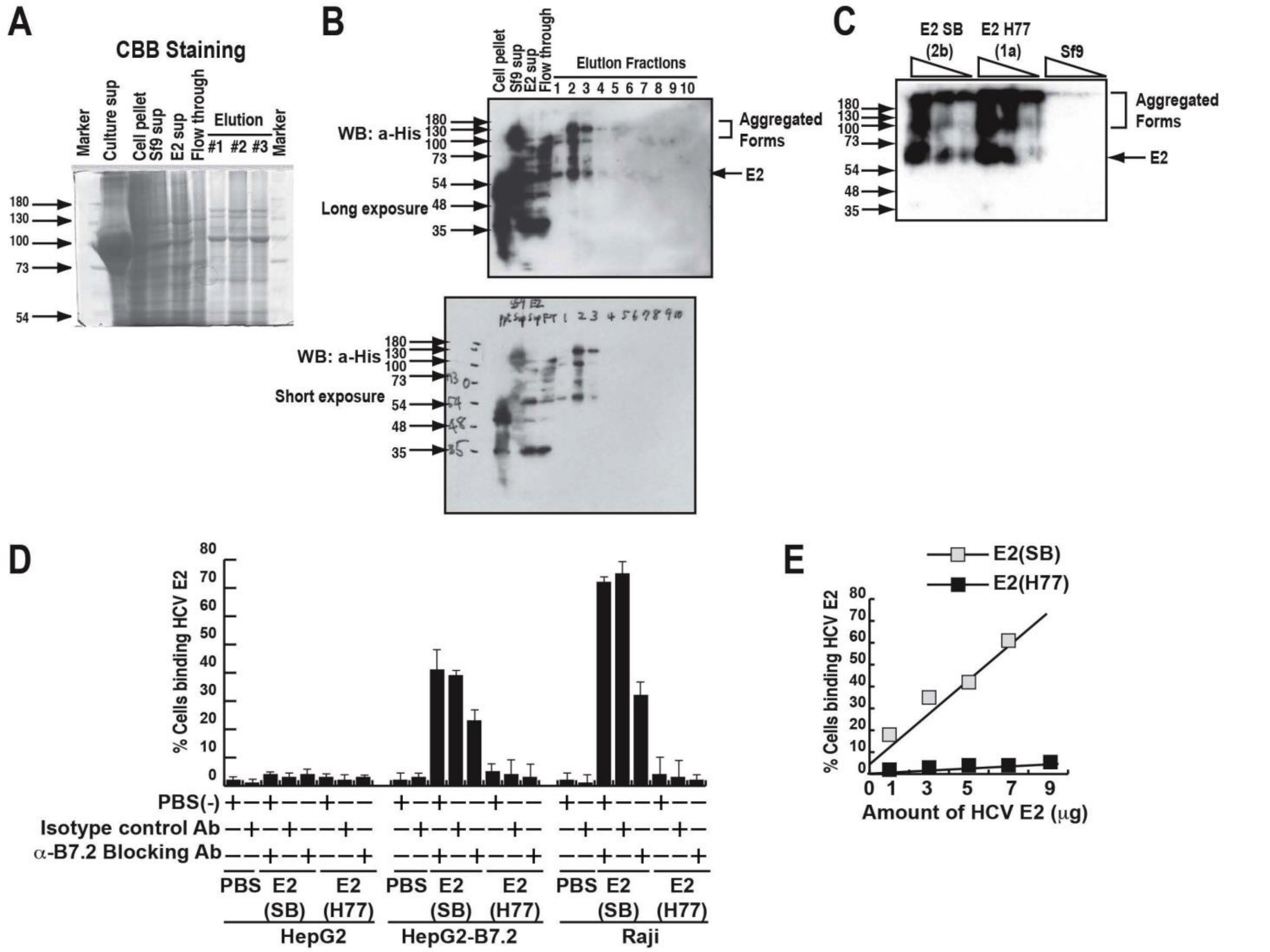

Supplementary Fig. 7. Recombinant HCV E2 ΔC-ter (SB strain), but not E2 ΔC-ter (H77 strain), purified from Baculovirus expression system binds cells through B7.2. (**A**) Coomassie Brilliant Blue (CBB) staining of sodium dodecyl sulfate-polyacrylamide (SDS-PAGE) gel for supernatant, flow-through fraction, washing fractions and elution fractions purified from Sf9 cells infected with recombinant Baculovirus expressing truncated E2 protein (truncation of cytoplasmic domain). Recombinant HCV E2661 was purified by Baculovirus expression system by using of His-tag. (**B**) Immunoblot analysis by using of anti-His antibody to detect His-tagged HCV E2 proteins purified from Baculovirus expression system. Monomeric and aggregated forms of E2 are indicated. (**C**) Lysates from Sf9 cells expressing HCV E2 proteins were separated by SDS-PAGE gel electrophoresis under non-reducing conditions. E2 was detected by immunoblotting with anti-E2 MAb. (**D**) Binding of E2 to Raji and HepG2-B7.2 cells in the presence or absence of a-B7.2 or isotype-matched control antibodies. The percentage of cells binding E2 was measured by FACS. Average values from two replicates are presented. Error bars represent standard deviation (n = 3, P<0.05). (**E**) Dose-response curve for E2 binding to Raji cells. Various amounts of E2 protein from genotypes 1a (H77 strain) and 2b (SB strain) were used in the binding experiment as in panel **D**.

Supplementary Figure 7
Chen et al.

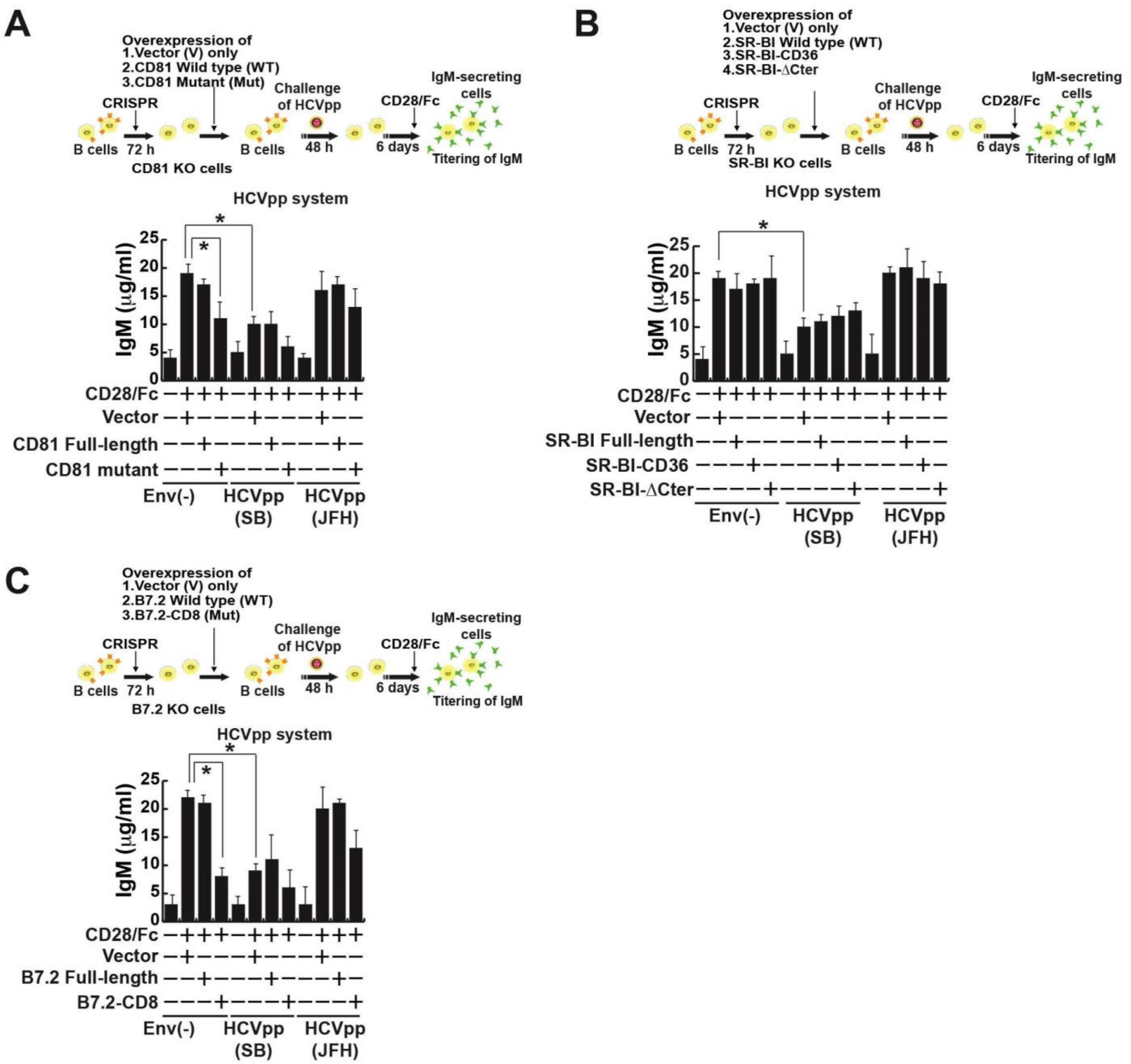

Supplementary Fig. 8. HCV entry into B cells reduces IgM production through downregulation of B7.2-CD28 signaling. (**A**) IgM ELISA assay. Wild-type CD81 (WT) or mutant CD81(Mut) was overexpressed in CD81-knockout (KO) Raji cells. After antibiotics selection, cells were infected with SB pseudo particle (SB HCVpp) for 24 hours. Infected cells then were harvested for qPCR assay. As shown in figure, overexpression of CD81 WT cells highly induced IgM expression, but not CD81 mutant cells. More interestingly, this phenomenon is abrogated in CD81 KO cells, implying that the synergistic interaction of CD81 with B7.2 is required for SB infection (n=3, **P*<0.05). (**B**) Wild-type (WT) or mutant (Mut) form in SR-BI expression vector was overexpressed in SR-BI or SR-BI-CD36 or SR-BI-C-terminus truncation mutant in SR-BI-knockout Raji cells. Error bars represent standard deviation (*n* = 3, **P*<0.05). (**C**) Wild-type (WT) or mutant form (B7.2-CD4 chimera receptor) of B7.2 was overexpressed in B7.2-knockout Raji cells. Note: B7.2-CD8 mutant oeverexpression failed to restore IgM production, indicating that cytoplasmic tail region (B7.2 signaling pathway) is required for IgM production. Error bars represent standard deviation (*n* = 3). **P*<0.05, by Student's *t*-test.

Supplementary Figure 8
Chen et al.

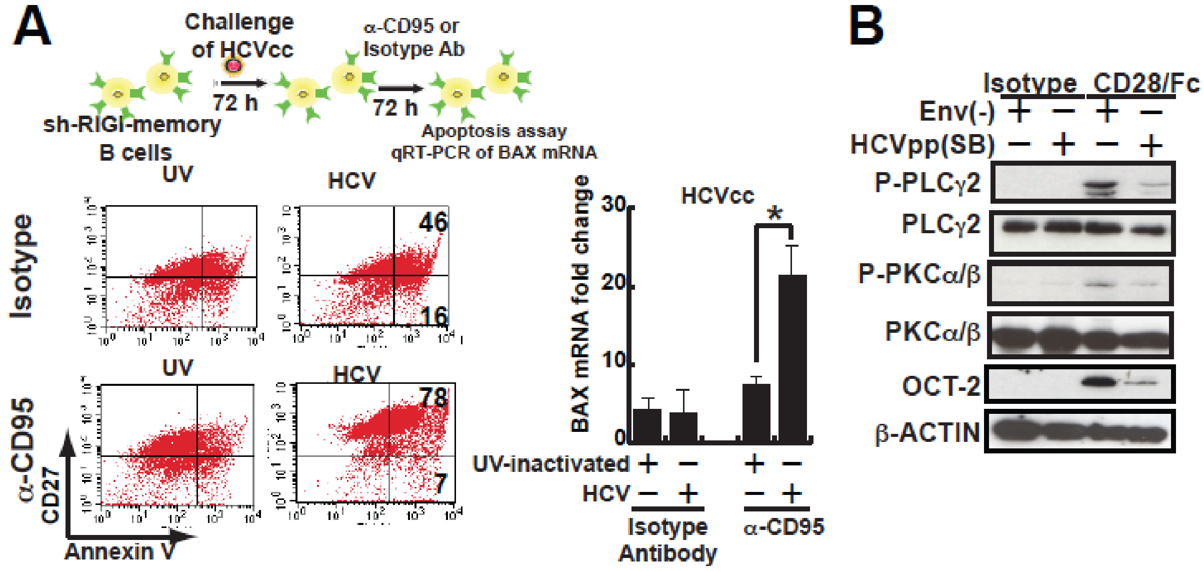

Supplementary Figure 9. HCV-infected memory B cells are prone to apoptosis and B7.2 stimulation increases a signaling cascade *in vivo*.

(**A**) HCV-infected memory B cells are prone to apoptosis. Memory B cells were incubated in the presence of media alone, mouse IgG1 (isotype control), or CD40L+IL-2+IL-10. Cells were then stained with annexin V and anti-CD27 mAbs and analyzed by flow cytometry (*$P<0.05$, by Student's *t*-test. n=3). Error bars represent S.D. (**B**) B7.2 stimulation increases a signaling cascade *in vivo*. Stimulation of B7.2 increased the phosphorylation PLCγ2 and PKCα/β and induction of OCT-2[1]. After resting B cells (5×10^5 cells/ml) were activated by CD40L/IL-2/IL-10 for 6 days, either CD28/Fc or a species- and isotype-matched control Ab (1 μg/ml) was added. Total proteins were examined by Western blot analysis to determine the level of OCT-2, and actin protein in the presence or absence of stimulation of B7.2[1].

Supplementary Figure 9
Chen et al.

Fig. 6A Left

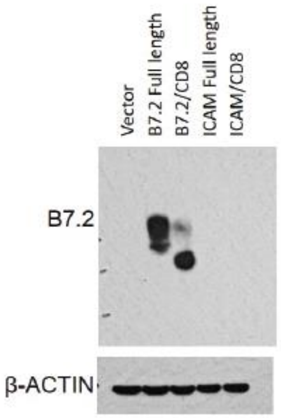

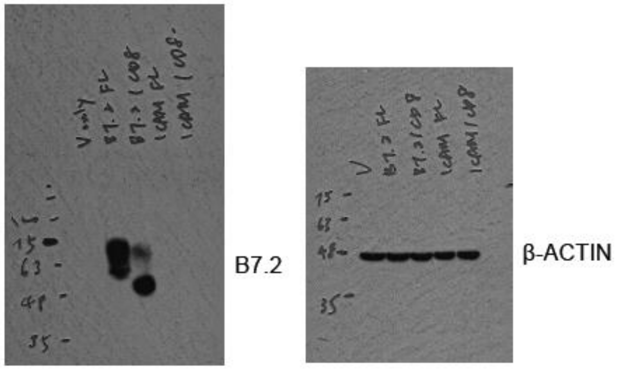

Fig. 6A Right

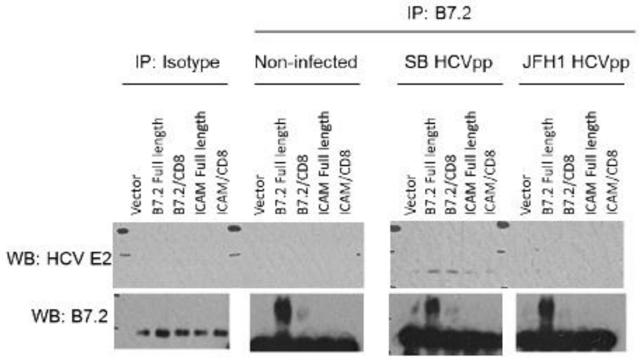

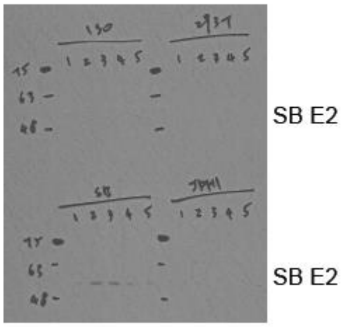

1. Vector only
2. B7.2 full length
3. B7.2/CD8
4. ICAM full length
5. ICAM/CD8

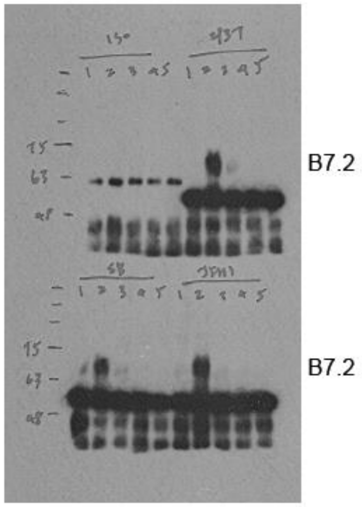

Supplementary Fig. 5C

IP: B7.2 →WB:E2

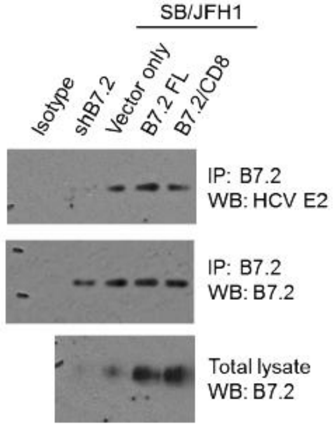

1. shB7.2 cells
2. SB/JFH1-V only in shB7.2 cells
3. SB/JFH1-B7.2 full length in shB7.2 cells
4. SB/JFH1-B7.2/CD8 in shB7.2 cells

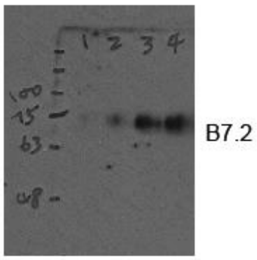

Supplementary Fig. 5C

1. Isotype control
2. shB7.2 cells
3. SB/JFH1-V only in shB7.2 cells
4. SB/JFH1-B7.2 full length in shB7.2 cells
5. SB/JFH1-B7.2/CD8 in shB7.2 cells

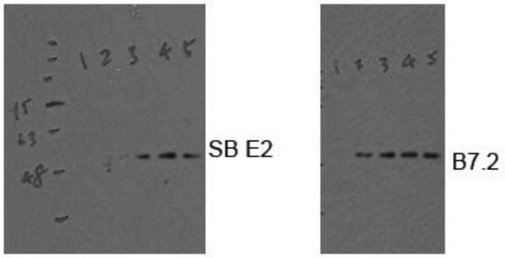

Supplementary Figure 10. Full blots figure.

Supplementary Figure 10
Chen et al.

Supplementary Note 1

Disulfide bonding between cysteine residues at 486 and 496 (in genotype 1: 494) maintains the structure and function of E2, indicating the essential role for this bond in hepatotropic viruses[2]. The G496 residue is located in part of CD28 or CTLA4 consensus binding motifs of B7.2 (MYPPPY)[3]. Cysteine to glycine substitution may open up this domain in the absence of disulfide bonding to allow binding to B7.2. Nucleotide substitution studies in sh-*RIGI*-Raji cells (**Fig. 3C**) indicated that nucleotides A181, U256, C276 and U280 of the 5'-UTR have a key role in regulating HCV levels in sh-*RIGI*-Raji cells. RNA stability, however, is not the only possibility. The base substitutions could affect mRNA translation/replication or viral replicase activity. More detailed study is warranted to identify the role of these residues in B7.2-E2 interactions.

Since HCV impairs the B7.2-mediated signaling pathway in infected B cells, memory B cells in HCV-infected patients may be affected by a bystander functional impairment in Ig class-switch. Indeed, one third of late onset hypogammaglobulinaemic patients, including common variable immunodeficiency (CVI), two hyper IgM syndromes (HIM) and two IgG subclass deficiency, had evidence of HCV infection[4]. Class switch, however, did not account for the decreased IgM in the subsets of HCV-infected individuals, as we consistently detected IgG in cell culture supernatants[47]. We tested class switching in HCV-infected hybridoma (B plasma cells), and showed that these cells did not undergo Ig class switching despite highly induced activation-induced cytidine deaminase (AID) levels[5]. Furthermore, from Dr. Rice's group there is evidence of functional impairment in HCV patients carrying HCV-infected B cells[6]. The evidence is consistent with the observation that HCV-infected individuals have lower levels of IgG production, although class switch recombination is not altered by HCV infection. The stimulation of B7.2 in CD40L/IL-4-activated B cells increases transcription of IgG1 by activating NF-κB[7]. This activation of NF-κB also increases activity of PI3K, which enhances phosphorylation of PDK1, AKT and IκB kinase αβ (IKKα/β)[7].

B7.2 expression levels were higher in dendritic cells, monocytes memory T cells and memory B cells, but was much less in naïve B cells and naïve T cells[8]. The B7.2 expression levels are partially correlated with levels of HCV replication (SB strain) since HCV RNA is detected in B and T lymphocytes, but not in DCs and moncytes in humanized mice and patients. Among immune cells, memory B cells and T cells support lymphotropic HCV SB strain replication, but monocytes and dendritic cells do not support HCV replication, indicating that additional unknown factors may be involved in HCV entry (e.g, CD5 as proposed in different systems)[9]. Dendritic cells have been reported to show detectable HCV RNA, but this evidence comes from particular cell structures (hepatocytes are extended inside of dendritic cells)[10]. Dr. Chisari's group has demonstrated that HCV-infected cells trigger a robust IFN response in plasmacytoid dendritic cells (pDCs) by a mechanism that requires active viral replication and Toll-like receptor 7 signaling in hepatocytes and direct cell-cell contact[11]. The same events are triggered by HCV subgenomic replicon cells but not by free virus particles, suggesting the existence of a novel cell-cell RNA transfer process (not viral particles) whereby HCV-infected cells can activate pDCs to produce IFN without infecting them[11].

Infection and replication of HCV in B cells is not uncommon in HCV-infected patients[12]. In fact, HCV has been detected in patient-derived B cells[13]. Therefore, HCV naturally infects B cells. In addition, HCV infection of human T cells has also been documented. To develop an HCV infection system in primary T cells and T cell lines, immune activation is required to upregulate the susceptibility of cells to both HCV infection and expression in infected cells[14,15]. The mechanism by which B7.2 downregulation on B cells (and other APC) may impair APC function in stimulating T cells warrants further investigation.

HCV infection induces type III IFN, which is dependent on the MDA5 (IPS-1) pathway in CD8(+) DCs and hepatocytes, leading to cytoplasmic antiviral protein expressions[16]. However, MDA5 silencing did not alter SB strain HCV RNA levels in Raji cells, indicating that SB HCV RNA may have mechanisms to escape the type III IFN-dependent pathway. Future study is required to fully understand the underlying mechanism of the type III IFN-dependent pathway.

Since DC-SIGN and CD81 are expressed on a subset of activated B cells and can bind to HCV of certain genotypes, these molecules may potentially complicate the studies described above. Further study is required to test if anti-DC-SIGN or anti-CD81 antibody can block or compete with E2 protein for the binding by HCV, to rule out non-specific effects that may be caused by DC-SIGN, CD81 or other unknown cell surface proteins.

Supplementary Methods

HCV clone

A previous study established the SB B-cell line from an HCV-infected non-Hodgkin's B-cell lymphoma isolated from a patient[17,18]. These cells are persistently infected with a HCV clone of genotype 2b, which we

termed SB-HCV17. Using 14 overlapping cDNA clones derived from SB cells, we constructed a plasmid containing the complete genome sequence of SB-HCV (pHCVSB2b). All amplified products were purified and then cloned into pGEM-T EASY vectors (Promega, Madison, WI), resulting in 14 plasmids, including pGEM1-399, pGEM45-1439, pGEM1137-1978, pGEM1618-2622, pGEM2430-3721, pGEM3585-4825, pGEM4600-5934, pGEM5365-6886, pGEM6412-7884, pGEM7714-8640, pGEM8351-9107, pGEM8854-9402, and pGEM9266-9695 (the numbers of each clone indicate the positions within SB: **Supplementary Fig. 1**). A promoter for T7 polymerase and an *XbaI* site were added to the clone by successive rounds of polymerase chain reaction (PCR) using primers with extended 5' termini. The full-length clone pHCV-SB2b thus contains a T7 promoter at the 5' terminus that allows transcription starting from the first G of the HCV genome. To determine the terminal 5' end sequence, cDNA was synthesized with a 5'UTR primer, tailed with terminal deoxynucleotydyl transferase and dCTP homopolymer, and then amplified by PCR (5'RACE system for Rapid Amplification of cDNA Ends Version 2.0, Invitrogen). To determine the HCV 3' sequences, 3'-blocked and 5'-phosphorylated oligonucleotide 206 was ligated to HCV genome RNA by using T4 RNA ligase.[19] Ligation reactions were used directly for cDNA synthesis. The dC-tailed cDNA was subjected to nested PCR by using primers G18XS and XS[20]. The amplified DNA fragment was cloned into the vector pGEMT-EASY. A mutant construct was derived from pHCVSB has a point mutation of this motif to GND (pHCVSB-GND), which abolishes the RNA polymerase activity of NS5B. The respective GND mutant RNAs were included. The consensus sequence of 20 isolates was adopted in each region. The secondary structure is predicted by MFOLD software as previously described[21]. The complete nucleotide sequence of the viable infectious molecular clone of the SB strain of HCV is deposited with GenBank (**The accession number: KM349851**).

To generate pSB/JFH1/GND chimera virus construct carrying a mutation in the NS5B GDD motif, which abolishes RNA polymerase activity, amino acid substitutions will be introduced by PCR-based site-directed mutagenesis in (MLVCGDDLVV) encompassing the GDD motif of NS5B, and amplified DNA fragments will be analyzed by automated nucleotide sequencing by using an ABI 310 sequencer. GDD motif will be changed into GND.

Infection of cells with secreted HCV

JFH-1 strain, SB strain from SB cells (spleen B cells), and SB/JFH1 chimera virus were used for *in vitro* infection studies[18,22]. Genotype 1a/1b hybrid strain was previously described[23]. Concentrated culture medium (100 µl) was used for inoculation of cells in a well of 12- or 6-well plate for 3 h with periodic rocking. At the end of inoculation, cells were washed 3 times with PBS, followed by addition of 1 or 2 ml of complete culture medium. At six days after infection, inoculated cells were fixed and subsequently stained using anti-NS3 antibodies. Positively stained cells were counted in a blinded fashion. HCV RNA titer was reduced by prior treatment with anti-E2 antibody. The titer of JFH1 viral stock was determined as previously described[22].

Real-Time RT-PCR

HCV RNA was quantified by real-time reverse transcription (RT)-PCR (RT-qPCR) with primers and the fluorogenic probe, labeled with 6-FAM and BHQ-1 (IDT, Inc.) using ABI 7900 Sequence Detector and TaqMan EZ RT-PCR core reagents (Applied Biosystems, Foster City, CA). The TaqMan primers and probe: (Forward) GACACTCCGCCATGAATCACT; (Reverse) GCCATGGCTAGACGCTTTCT; (Probe) CCCTGTGAGGAACTAC. The real time PCR signals were analyzed using SDS software (Version 2.1; Applied Biosystems). For miR-122 expression analysis, RNA was reversed transcribed by High-Capacity cDNA synthesis Kit (Applied Biosystems) and normalized by U6 RNA levels as previously described[24].

Negative strand detection of HCV

Negative strand-specific SB-HCV RNA was detected by using of a nested PCR method using 4-fold serial dilution of RNA by using a recently established procedure previously described[25-27]. Viral RNA was purified with QIAmp Viral RNA Minikit (Qiagen). Negative strand–specific SB-HCV RNA were detected by use of a nested polymerase chain reaction (PCR) method

RNA transfection

For Raji and sh-*RIGI*-Raji cells, RNA (10 µg) was mixed with 400 µl of Raji cell suspension and pulsed at 260 V and 975 µF with the Gene Pulser II (Bio-Rad, Hercules, CA). For Huh7.5 or Huh7.5.1 cells, synthesized RNA was used for electroporation as previously described[22].

Generation of HCVpp

To determine whether HCV pseudo-particles are assembled *in vitro*, HCV envelope proteins were expressed from a single polyprotein precursor and individually released in their respective cell compartments on cleavage by cellular and viral proteases[28]. The expression vectors encoding the E1 and E2 glycoproteins from several genotypes of HCV (1b, 2a or 2b) were generated by inserting a DNA fragment encoding the last 60 residues of HCV core and all of the E1 and E2 proteins into a non-packageable, CMV promoter-driven expression construct pCDNA3.1[29]. To produce virus pseudo-types, HIV pseudo-types were generated by cotransfection of 2.5×10^6 HEK293T cells were co-transfected in 10 cm-plates with a packaging-competent plasmids encoding an envelope-defective HIV-1 proviral genome and the luciferase reporter in envelope-defective pNL4.3.Luc.R-E- proviral genome (NL4.3.Luc.R^-E^-)[29,30], plasmids expressing the viral glycoproteins (pCMV-HCV E1E2p7 or pCMV-HCV E1E2), including SB (genotype 2b), N (N E1E2), and strain Con1 HCV E1E2 gps or an empty vector. All virus stocks were normalized for p24 HIV core antigen and infected at 1 ng per well, with the exception of VSV gp and MLV gp, which was used at 0.01 ng per well. Positive control pseudo-particles were generated without glycoproteins and with the VSV-G (pMD.G)[29] or Murine Leukemia Virus (MLV) envelope glycoprotein expression vectors. The medium (8 ml/plate) was replaced 16 h after transfection. Target cells were seeded in 12-well plates at a density of 8×10^4 cells per well and incubated overnight at 37°C.

Assessment and titration of HCVpp

HIV p24 proteins were detected as Gag precursors of 24 kD. Viral particles were harvested from the supernatant of transfected cells and purified by ultracentrifugation through high density sucrose cushions, a purification process that removes loosely associated glycoproteins[29].

Synthesis of miR-122 antagomirs

The single-stranded RNAs and modified RNA analogues consisted of a 21–23-nucleotide length with modifications as previously described[31]: anti-122, 5'-acaaacaccauugucacacucca-3'; anti-122pS, 5'-ascsaaacaccauugucacacsuscscsa-3'; anti-122fS, 5'-ascsasasascsascscsasususgsuscsascsascsuscscsa-3'; antagomir-122, 5'-a$_s$c$_s$aaacaccauugucacacusc$_s$c$_s$a$_s$-Chol-3'; mm-antagomir-122, 5'-a$_s$c$_s$acacaacacugucacauu$_s$c$_s$c$_s$a$_s$-Chol-3'

Persistent HCV infection of humanized mouse model

To further study the generality of the HCV infection of human immune systems, we developed a mouse model grafted with the human CD34+ hematopoietic cells. In this model, immunocompromised RG mice (*Rag2-/-;Il2rγC-/-*: Jackson Laboratories) or AFC8 transgenic mice[32] were bred and maintained under pathogen-free conditions. All experiments involving animals were conducted at University of North Carolina in accordance with guidelines established by the Animal Welfare Act and the NIH Guide for the Care and Use of Laboratory Animals. Animal facilities at University of North Carolina are fully accredited by the Association for Assessment and Accreditation of Laboratory Animal Care International (AAALAC). The Institutional Animal Care and Use Committee (IACUC) of University of North Carolina approved all animal studies. Human CD34+ cells were injected intravenously into 5-day-old-RG mice. The persistence of human mononuclear cells in these mice was confirmed by testing cell preparations for human CD45 positivity by FACS and human HLA DNA positivity by PCR using human HLA-specific primers. Sera collected from these mice at the same time points were negative for HLA DNA, demonstrating the absence of human DNA contamination. RG or AFC8 mice inoculated with sheep PBMC were used as a negative control; all cell and serum samples from these mice were negative for human HLA DNA sequences. One week after human mononuclear cell grafting, when a high level of human peripheral blood leukocytes was first reached, the humanized RG-hu-HSC mice were intravenously injected with cell culture-derived HCV-SB virus, HCV-JFH1 virus or a chimera SB-JFH1 virus, which contains the SB E1E2 genes in the JFH1 genomic backbone. The HCV RNA levels in these mice were then analyzed by real-time RT-PCR at several different time points sequentially.

Exosome Isolation

Exosome was isolated from plasma from HCV patients and healthy individuals by ExoQuick exosome precipitation solution and centrifuged at 1,500*g* for 30 minutes from 100 µl of plasma after incubation at 4°C for 30 minutes (particle size: up to 90 nm) in accordance with the manufacturer's protocol (SBI System Biosciences: Cat. #EXOQ5A) as previously described[19,20]. Informed consent and an Institutional IRB

committee approval was obtained in University of Southern California. The supernatant was denoted the protein-rich fraction, whereas the pellet was denoted the exosome-rich fraction. The pellet (the exosome-rich fraction) was washed twice with phosphate-buffered saline (PBS) and lysed with QIAzole (Qiagen) or lysed with TRI reagent for total RNA or protein isolation. As the exosome precipitation solution precipitates up to 90 nm size particles, microparticles (≈200-1,000 nm) is in protein-rich fraction.

Plasma MicroRNA Analysis

Re-suspend the UniSp6 RNA spike-in (from the Universal cDNA synthesis kit II, #203301, EXIQON, Inc.) by adding 80 µl nuclease free water to the vial. Re-suspend the U6 RNA or cel-miR-39-3p RNA spike-in to the vial. After leave for 20-30 min on ice, solution was mixed by vortexing and spinning down. Store in aliquots at -20°C. Prior to the RT reaction, add 1 µL synthetic spike-in mix per 20 µl cDNA synthesis. After equal volumes of plasma or serum samples were thawed on ice, plasma miR was isolated as previously described[33,34] using TRI reagent and measured by TaqMan miRNA Assay (Applied Biosystems). Synthetic U6 RNA or *Caenorhabditis elegans* (cel)-miR-39 was spiked and after this step miRNeasy kit protocol was followed as per instructions (Qiagen). Cel-miR-39 was used to normalize the technical variation between the samples.

Coreceptor cloning by lentivirus Raji cell cDNA library screen in HEK293T cells

To identify the possible novel receptor specific for the infection of lymphotropic HCV, We performed a lentivirus based screen of a cDNA library, derived from the highly HCV-permissive B lymphoma cell lines Raji, for genes that render the non-permissive $CD81^+$ $SR\text{-}BI^+$ HEK293T cell line infectable with HIV-1 particles pseudo-typed with HCVgp (HCVpp). A cDNA expression library (pLIB-Raji) was generated by reverse-transcribing mRNA from the highly HCV-permissive B lymphoma cell lines Raji cells using SuperScript Full-length cDNA Library Construction Kit Core Module (A11181-02: Invitrogen). A lentiviral cDNA library, creating targeting vectors harboring cDNAs, and cloning them into lentivirus by the Gateway recombination method using ViraPower™ Lentiviral Gateway Expression System (Invitrogen) by following instruction manual. This cDNA library was incorporated into the genome of pseudo-type lentivirus virions containing VSV-G protein as the envelope protein. HEK293T cells were first transduced with lentiviral cDNA library and then challenged with a selectable HCV pseudo-type virus (pHIV-puro) in which SB-HCV envelope proteins package an *env*-negative HIV-1 provirus containing the puromycin resistant gene. Cells were treated with puromycin two days after infection. Under this condition, only the cell expressing the lymphotropic receptor susceptible to SB-HCV could be infected by this pseudo-type virus and selected by puromycin treatment. After selection, cell viability was quantified by Trypan Blue exclusion. As a result of this screen, ten candidate receptors were identified as a potential HCV entry factor. As three negative controls, parental HEK293T cells were challenged with (i) no pseudo-virions or (ii) with HCV-puro pseudo-types. (iii) Library-transduced cells were challenged with no pseudo-virions.

E2 binding assays

Binding of E2 to cell surface via B7.2 was analyzed by a fluorescence-activated cell sorting (FACS)-based assay as previously described[35,36]. Approximately 2×10^5 cells were washed twice in PBS-1% fetal calf serum (FACS buffer) and incubated with the partially purified E2 (10 µg) at room temperature for 1 h. After washing, a His probe (Santa Cruz Biotechnology) was added to the mixture at 2 µg/ml and incubated for 1 h at r.t. Cell-bound His-probe was detected with anti-rabbit IgG-fluorescein isothiocyanate conjugate (Jackson Immunoresearch laboratories, West Grove, Pa.). Flow cytometry was performed on a FACSCalibur flow cytometer (Becton Dickinson, San Jose, Calif.). For binding inhibition assays, cells were incubated in FACS buffer containing 20 µg of anti-B7.2 (Santa Cruz Biotechnology) per ml for 30 min at r.t. prior to incubation with E2. To test the relative binding efficiency, a dose-response curve of E2 was determined. The percent cells binding E2 was derived from the best-fit analysis in the linear range of each curve.

Calcium mobilization assay

Calcium mobilization assay was performed as previously described[6]. After B cells (2×10^6) were incubated with 1µM Indo-1 for 30 minutes, cells were labeled with anti-CD27 and AnnexinV and suspended in HBSS containing Ca^{2+} and 1% BSA. After addition of 10 µg/mL goat $F(ab')_2$ anti-IgM, emission at 405 and 495 nm was measured with FACSCaliber and 405/495 nm emission ratios of IgM^- B-cell were calculated.

Annexin V apoptosis assay

Annexin V apoptosis assay was performed as previously described[6]. The cells were fixed in cold 70% ethanol (0.5 ml) at 4 °C for 1 h. Memory B cells (2 × 10^6) were incubated with 1 μg/mL FLAG-tagged CD40L, 6 U/mL IL-2 (R&D Systems) and 200 ng/mL IL-10 (R&D Systems) with 2 μg/mL mouse IgG_1 anti-FLAG antibody (Alexis Biochemicals) in RPMI/10% fetal calf serum at 37°C for 0 and 6 hours and were washed with PBS and resuspended. After incubation with annexin V–phycoerythrin at r.t., cells were stained with anti-CD27 FITC at r.t. Flow cytometry was performed. Ratio of apoptosis was examined by a FACScan data acquisition for red fluorescence.

The secretion of IgM isotypes

To test whether binding by HCV to B7.2 can downregulates B7.2-induced signaling pathways, immunoglobulin secretion assays were performed as previously described[6]. Resting B cells (50,000/well in 96-well round-bottom plates) were were purified from PBMC by negative selection and cultured for 6 days with 1 μg/mL FLAG-tagged CD40L, 6 U/mL IL-2 (R&D Systems) and 200 ng/mL IL-10 (R&D Systems) with 2 μg/mL mouse IgG_1 anti-FLAG antibody (Alexis Biochemicals) in RPMI (10% fetal calf serum, 2mM L-glutamine, 100 U/mL penicillin/streptomycin, and 0.25 μg/mL amphotericin B). Then, memory B cells were stimulated with CD28/Fc fusion protein for 30 minutes after incubation with CD28/Fc for immunoblot analysis. To determine whether PLCγ2 is activated in B cells by HCVpp-SB, P-PLCγ2 was examined by immunoblot with the anti-P-PLCγ2 antibody. Cells treated with human CD28/Fc fusion protein (R&D Systems), which has been shown to induce B7.2 signaling[7]. For ELISPOT, memory B cells were stimulated with CD28/Fc fusion protein for 48 h and washed with RPMI, placed on MultiScreen filter plates (Millipore), coated with goat $F(ab')_2$ anti–human IgM (Jackson ImmunoResearch Laboratories), and incubated at 37°C for 6 hours. Plates were then incubated with horseradish peroxidase-labeled anti–human IgM, and the assays were developed with 3-amino-9-ethylcarbazole (Sigma-Aldrich) and spots were counted. For ELISA, the supernatants were collected on day 6 and analyzed for IgM by enzyme-linked immunosorbent assay[37]. Briefly, wells were coated overnight at 4 °C with 5 μg/ml goat anti-mouse IgM Abs in carbonate-bicarbonate buffer, pH 9.6. The unbound sites were blocked with 2% BSA, and then culture supernatants were added for 2 h at 37 °C and incubated with rabbit anti-mouse IgM horseradish peroxidase-labeled antibody at 37 °C for 2 h. Plates were then incubated with HRP-labeled goat-anti–human IgM, and the assays were developed with TMB (BioFX Laboratories). After stopping reactions with 1N H_2SO_4, A_{450} was measured on a μQuant microplate reader (BIO-TEK Instruments, Inc.). The concentration of Abs is represented as nanograms/ml, as computed by using standard human IgM isotypes.

Statistical analysis

ANOVA analysis was used for multiple comparisons. Pearson's correlation test was performed for correlation analysis in GraphPad Prism software. For all other statistical analyses, the nonparametric Mann-Whitney test or two-tailed t test was employed. Values of $p < 0.05$ were considered as statistically significant.

Supplementary References

1 Podojil, J. R., Kin, N. W. & Sanders, V. M. CD86 and beta2-adrenergic receptor signaling pathways, respectively, increase Oct-2 and OCA-B Expression and binding to the 3'-IgH enhancer in B cells. *The Journal of biological chemistry* **279**, 23394-23404, doi:10.1074/jbc.M313096200 (2004).

2 McCaffrey, K. *et al.* Role of conserved cysteine residues in hepatitis C virus glycoprotein e2 folding and function. *Journal of virology* **86**, 3961-3974, doi:10.1128/JVI.05396-11 (2012).

3 Sharpe, A. H. & Freeman, G. J. The B7-CD28 superfamily. *Nat Rev Immunol* **2**, 116-126, doi:10.1038/nri727 (2002).

4 Quinti, I. *et al.* HCV infection in patients with primary defects of immunoglobulin production. *Clin Exp Immunol* **102**, 11-16 (1995).

5 Machida, K. *et al.* Hepatitis C virus (HCV)-induced immunoglobulin hypermutation reduces the affinity and neutralizing activities of antibodies against HCV envelope protein. *Journal of virology* **82**, 6711-6720, doi:10.1128/JVI.02582-07 (2008).

6 Charles, E. D. *et al.* Clonal B cells in patients with hepatitis C virus-associated mixed cryoglobulinemia contain an expanded anergic CD21low B-cell subset. *Blood* **117**, 5425-5437, doi:10.1182/blood-2010-10-312942 (2011).

7 Kin, N. W. & Sanders, V. M. CD86 stimulation on a B cell activates the phosphatidylinositol 3-kinase/Akt and phospholipase C gamma 2/protein kinase C alpha beta signaling pathways. *Journal of immunology* **176**, 6727-6735 (2006).

8 Hakamada-Taguchi, R. *et al.* Expression and co-stimulatory function of B7-2 on murine CD4+ T cells. *Eur J Immunol* **28**, 865-873, doi:10.1002/(SICI)1521-4141(199803)28:03<865::AID-IMMU865>3.0.CO;2-T (1998).

9 Curry, M. P. *et al.* Expansion of innate CD5pos B cells expressing high levels of CD81 in hepatitis C virus infected liver. *Journal of hepatology* **38**, 642-650 (2003).

10 Takahashi, K. *et al.* Plasmacytoid dendritic cells sense hepatitis C virus-infected cells, produce interferon, and inhibit infection. *Proceedings of the National Academy of Sciences of the United States of America* **107**, 7431-7436, doi:10.1073/pnas.1002301107 (2010).

11 Dreux, M. *et al.* Short-range exosomal transfer of viral RNA from infected cells to plasmacytoid dendritic cells triggers innate immunity. *Cell Host Microbe* **12**, 558-570, doi:10.1016/j.chom.2012.08.010 (2012).

12 Pham, T. N. *et al.* Hepatitis C virus replicates in the same immune cell subsets in chronic hepatitis C and occult infection. *Gastroenterology* **134**, 812-822, doi:10.1053/j.gastro.2007.12.011 (2008).

13 Cheng, J. L. *et al.* Hepatitis C virus in human B lymphocytes transformed by Epstein-Barr virus in vitro by in situ reverse transcriptase-polymerase chain reaction. *World journal of gastroenterology* **7**, 370-375 (2001).

14 Pham, T. N. *et al.* Mitogen-induced upregulation of hepatitis C virus expression in human lymphoid cells. *J Gen Virol* **86**, 657-666, doi:10.1099/vir.0.80624-0 (2005).

15 MacParland, S. A., Pham, T. N., Gujar, S. A. & Michalak, T. I. De novo infection and propagation of wild-type Hepatitis C virus in human T lymphocytes in vitro. *J Gen Virol* **87**, 3577-3586, doi:10.1099/vir.0.81868-0 (2006).

16 Okamoto, M. *et al.* IPS-1 is essential for type III IFN production by hepatocytes and dendritic cells in response to hepatitis C virus infection. *Journal of immunology* **192**, 2770-2777, doi:10.4049/jimmunol.1301459 (2014).

17 Levine, A. M., Shimodaira, S. & Lai, M. M. Treatment of HCV-related mantle-cell lymphoma with ribavirin and pegylated interferon Alfa. *The New England journal of medicine* **349**, 2078-2079 (2003).

18 Sung, V. M. *et al.* Establishment of B-cell lymphoma cell lines persistently infected with hepatitis C virus in vivo and in vitro: the apoptotic effects of virus infection. *Journal of virology* **77**, 2134-2146 (2003).

19 Kolykhalov, A. A., Feinstone, S. M. & Rice, C. M. Identification of a highly conserved sequence element at the 3' terminus of hepatitis C virus genome RNA. *Journal of virology* **70**, 3363-3371 (1996).

20 Tanaka, T., Kato, N., Cho, M. J. & Shimotohno, K. A novel sequence found at the 3' terminus of hepatitis C virus genome. *Biochem Biophys Res Commun* **215**, 744-749 (1995).

21 Honda, M., Beard, M. R., Ping, L. H. & Lemon, S. M. A phylogenetically conserved stem-loop structure at the 5' border of the internal ribosome entry site of hepatitis C virus is required for cap-independent viral translation. *Journal of virology* **73**, 1165-1174 (1999).

22 Wakita, T. *et al.* Production of infectious hepatitis C virus in tissue culture from a cloned viral genome. *Nature medicine* **11**, 791-796 (2005).
23 Choi, J. *et al.* Reactive oxygen species suppress hepatitis C virus RNA replication in human hepatoma cells. *Hepatology* **39**, 81-89 (2004).
24 Urban, T. J. *et al.* IL28B genotype is associated with differential expression of intrahepatic interferon-stimulated genes in patients with chronic hepatitis C. *Hepatology* **52**, 1888-1896 (2010).
25 Hu, Y., Shahidi, A., Park, S., Guilfoyle, D. & Hirshfield, I. Detection of extrahepatic hepatitis C virus replication by a novel, highly sensitive, single-tube nested polymerase chain reaction. *Am J Clin Pathol* **119**, 95-100 (2003).
26 Negro, F. *et al.* Detection of genomic- and minus-strand of hepatitis C virus RNA in the liver of chronic hepatitis C patients by strand-specific semiquantitative reverse-transcriptase polymerase chain reaction. *Hepatology* **29**, 536-542 (1999).
27 Kondo, Y., Sung, V. M., Machida, K., Liu, M. & Lai, M. M. Hepatitis C virus infects T cells and affects interferon-gamma signaling in T cell lines. *Virology* **361**, 161-173 (2007).
28 Bartenschlager, R. & Lohmann, V. Replication of hepatitis C virus. *J Gen Virol* **81**, 1631-1648 (2000).
29 Negre, D. *et al.* Lentiviral vectors derived from simian immunodeficiency virus. *Curr Top Microbiol Immunol* **261**, 53-74 (2002).
30 Bartosch, B., Dubuisson, J. & Cosset, F. L. Infectious hepatitis C virus pseudo-particles containing functional E1-E2 envelope protein complexes. *The Journal of experimental medicine* **197**, 633-642 (2003).
31 Krutzfeldt, J. *et al.* Silencing of microRNAs in vivo with 'antagomirs'. *Nature* **438**, 685-689, doi:10.1038/nature04303 (2005).
32 Washburn, M. L. *et al.* A humanized mouse model to study hepatitis C virus infection, immune response, and liver disease. *Gastroenterology* **140**, 1334-1344, doi:10.1053/j.gastro.2011.01.001 (2011).
33 Bala, S. *et al.* Circulating microRNAs in exosomes indicate hepatocyte injury and inflammation in alcoholic, drug-induced, and inflammatory liver diseases. *Hepatology* **56**, 1946-1957, doi:10.1002/hep.25873 (2012).
34 Kroh, E. M., Parkin, R. K., Mitchell, P. S. & Tewari, M. Analysis of circulating microRNA biomarkers in plasma and serum using quantitative reverse transcription-PCR (qRT-PCR). *Methods* **50**, 298-301, doi:10.1016/j.ymeth.2010.01.032 (2010).
35 Flint, M. *et al.* Characterization of hepatitis C virus E2 glycoprotein interaction with a putative cellular receptor, CD81. *Journal of virology* **73**, 6235-6244 (1999).
36 Machida, K., Cheng, K. T., Pavio, N., Sung, V. M. & Lai, M. M. Hepatitis C virus E2-CD81 interaction induces hypermutation of the immunoglobulin gene in B cells. *Journal of virology* **79**, 8079-8089, doi:10.1128/JVI.79.13.8079-8089.2005 (2005).
37 Agrewala, J. N., Suvas, S., Verma, R. K. & Mishra, G. C. Differential effect of anti-B7-1 and anti-M150 antibodies in restricting the delivery of costimulatory signals from B cells and macrophages. *Journal of immunology* **160**, 1067-1077 (1998).
